# SARA suppresses myofibroblast precursor transdifferentiation in fibrogenesis in a mouse model of scleroderma

**DOI:** 10.1172/jci.insight.160977

**Published:** 2022-11-08

**Authors:** Katia Corano Scheri, Xiaoyan Liang, Vidhi Dalal, I. Caroline Le Poole, John Varga, Tomoko Hayashida

**Affiliations:** 1 Department of Pediatrics, Feinberg School of Medicine, Northwestern University, Chicago, Illinois, USA.; 2 Pediatric Nephrology, Ann and Robert H. Lurie Children’s Hospital of Chicago, Chicago, Illinois, USA.; 3 Departments of Dermatology and Microbiology and Immunology, Feinberg School of Medicine, Northwestern University, Chicago, Illinois, USA.; 4 Division of Rheumatology, Department of Internal Medicine, University of Michigan, Ann Arbor, Michigan, USA.

**Keywords:** Cell Biology, Fibrosis, Pericytes, Skin

## Abstract

We previously reported that Smad anchor for receptor activation (SARA) plays a critical role in maintaining epithelial cell phenotype. Here, we show that SARA suppressed myofibroblast precursor transdifferentiation in a mouse model of scleroderma. Mice overexpressing *SARA* specifically in PDGFR-β^+^ pericytes and pan-leukocytes (*SARATg*) developed significantly less skin fibrosis in response to bleomycin injection compared with wild-type littermates (*SARAWT*). Single-cell RNA-Seq analysis of skin PDGFR-β^+^ cells implicated pericyte subsets assuming myofibroblast characteristics under fibrotic stimuli, and *SARA* overexpression blocked the transition. In addition, a cluster that expresses molecules associated with Th2 cells and macrophage activation was enriched in *SARAWT* mice, but not in *SARATg* mice, after bleomycin treatment. Th2-specific *Il-31* expression was increased in skin of the bleomycin-treated *SARAWT* mice and patients with scleroderma (or systemic sclerosis, SSc). Receptor-ligand analyses indicated that lymphocytes mediated pericyte transdifferentiation in *SARAWT* mice, while with *SARA* overexpression the myofibroblast activity of pericytes was suppressed. Together, these data suggest a potentially novel crosstalk between myofibroblast precursors and immune cells in the pathogenesis of SSc, in which SARA plays a critical role.

## Graphical abstract

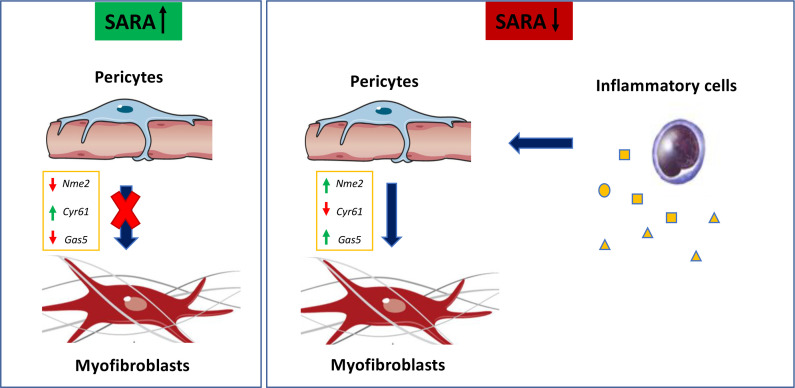

## Introduction

Scleroderma or systemic sclerosis (SSc) is an autoimmune disease of unclear etiology, characterized by cutaneous and visceral fibrosis. It is often fatal due to systemic nature of the disease, with particularly severe manifestations in the lungs (1).

Unfortunately, therapeutic approaches currently available for SSc are mostly nonspecific, creating off-target toxicity with little efficacy (2, 3). Most current approaches mainly target immune cells, and only a few target signals in activated ECM-producing myofibroblasts, such as nintedanib, which is an antifibrotic drug (4, 5), with limited successes. Instead, the following 2 novel strategies that target earlier pathogenic changes in SSc are novel and promising (6) to control this devastating disease more effectively. The first is to prevent transdifferentiation of myofibroblast precursors, and the second involves suppressing a specific subset of immune cells that contribute to disease progression. Indeed, the cytokines and chemokines detected in patients with SSc not only drive inflammation, but also enhance fibrogenesis by stimulating myofibroblast precursors (7–9), implicating that these 2 novel approaches are related and potentiate each other.

To establish these novel therapeutic approaches, first, the identity of myofibroblast precursors and molecular mechanisms by which those precursors undergo transdifferentiation to myofibroblasts need to be defined. Recent evidence suggests that pericytes are a major source of myofibroblasts in fibrogenesis (10–13). While blood vessel–supporting cells that surround capillaries, precapillary arterioles, and postcapillary venules are collectively called pericytes, several types of pericytes that are different in their morphology, function, and location within the vascular network exist (14, 15). Their main physiological function is to maintain vascular homeostasis (16–19). In addition, under stress or after injury, pericytes can undergo functional and phenotypic changes and contribute to pathological conditions (20–25), further supporting the assumption that pericytes, or at least some of them, can be the precursors for myofibroblasts.

We previously reported that Smad anchor for receptor activation (SARA) is essential for maintaining the epithelial cell phenotype (26). Despite SARA being originally reported as an adaptor for the transforming growth factor-β (TGF-β) receptor (27, 28), we and others subsequently reported that SARA is dispensable for TGF-β signaling and exerts functions that are not directly associated with TGF-β pathway, such as in intracellular molecular trafficking, cellular phenotype maintenance, and neuronal development (29–33). Interestingly, reanalysis of publicly available National Center for Biotechnology Information Gene Expression Omnibus (NCBI GEO) database data sets showed that SARA is detected in normal skin tissue and decreased in skin samples from patients with SSc (GSE9285 reported in ref. 34) and in cultured fibroblasts treated with fibrotic stimuli (GSE27165 reported in ref. 35). Our in vitro studies suggested that low SARA levels lead to spontaneous acquisition of mesenchymal phenotype in epithelial cells, implicating loss of SARA as one of the initial events leading to myofibroblast precursor transdifferentiation (26).

Together, these findings led us hypothesize that SARA regulates myofibroblast precursor transdifferentiation, and we tested the hypothesis using a mouse model that overexpresses *SARA* specifically in pericytes (36, 37).

## Results

### Effects of SARA overexpression in PDGFR-β^+^ cells on histological changes in a mouse SSc model.

**Figure 1:**
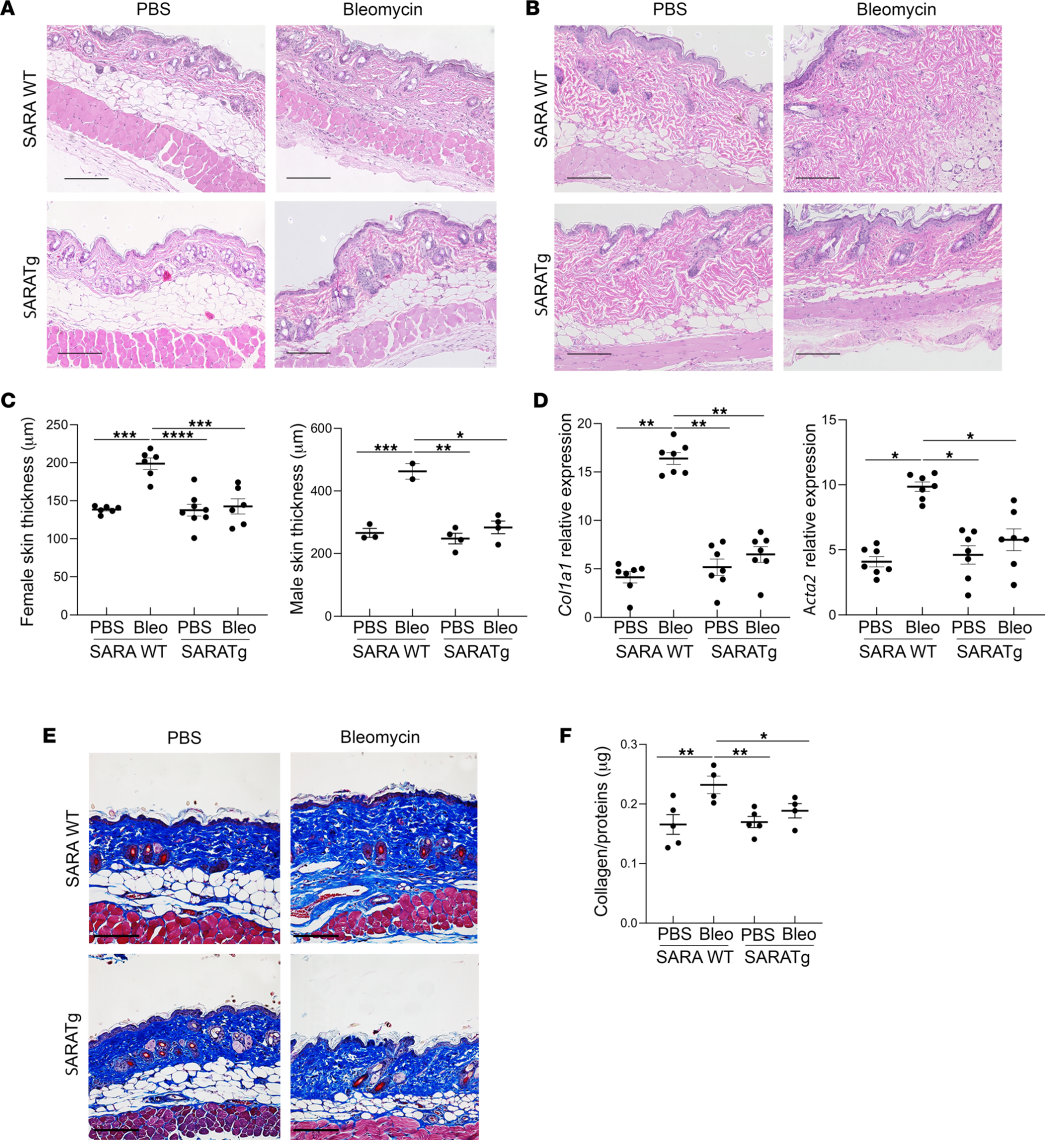
Effect of *SARA* overexpression in suppressing skin fibrosis in a mouse model of SSc. Representative images of hematoxylin-eosin (H&E) staining of the mouse skin from females (**A**) and males (**B**) subjected to PBS (left) or bleomycin (right) are shown. Dermal thickness is shown separately for female and male samples in the graphs (**C**). Each dot represents the value from a different mouse, and the average ± SEM for each condition is overlaid. mRNA expression for profibrotic gene Col1*a*1 and for activated myofibroblast marker *Acta2* are shown (**D**). Masson’s trichrome staining and collagen protein deposition in skin are shown (**E** and **F**). Scale bar = 100 μm. *SARAWT* mice *n* = 14 (PBS treated *n* = 7 and bleomycin treated *n* = 7) versus *SARATg* mice *n* = 15 (PBS treated *n* = 7 and bleomycin treated *n* = 7). One-way ANOVA followed by Tukey’s multiple comparisons test: *P < 0.05, **P < 0.01, ***P < 0.001, and ****P < 0.0001.

Pericytes are defined by their anatomical location (38); however, their morphology and functions are diverse. To date, no specific surface markers that reflect their physiological diversity have been defined to our knowledge. The most common antigens used to identify pericytes are the platelet-derived growth factor receptor-β (PDGFR-β) together with the proteoglycan neurin glial antigen 2 (NG2), which is a coreceptor for PDGFR-β (39). We generated mice that overexpress *SARA* specifically in PDGFR-β^+^ cells (*PDGFRb-Cre* *SARATg* mice, *SARATg* hereafter) (40) (Supplemental Figure 1; supplemental material available online with this article; https://doi.org/10.1172/jci.insight.160977DS1), then subjected them and their WT littermates (*PDGFRb-Cre* *SARA^–/–^* mice, *SARAWT* hereafter) to repeated subcutaneous injections of bleomycin, a widely used method to model SSc in mice (41–43). After 2 weeks of treatment, *SARAWT* mice developed a fibrotic phenotype, as detected by histological evaluation of the skin (Figure 1, A and B). In healthy *SARAWT* animals, as has been reported (44), male mice had significantly thicker dermal and thinner hypodermal skin layers compared with female mice (Figure 1C, *SARAWT* females treated with PBS in the left graph and males in the right graph). Therefore, we analyzed dermal thickness separately for male and female animals. Despite the differences, *SARAWT* animals treated with PBS as a vehicle showed a normal stratification of skin layers, whereas *SARAWT* mice treated with bleomycin showed a reduction of the adipose layer and a significant increase in dermal thickness. In contrast, the dermis of the *SARATg* mice was significantly less thick, and the fat layer was preserved in both males and females, even after bleomycin treatment (Figure 1C).

### Effects of SARA overexpression on profibrotic gene expression levels and collagen deposition.

One of the main events in fibrogenesis is the unbalanced deposition of ECM components, which compromises tissue plasticity (45). Bleomycin-treated *SARAWT* mice showed a significant increase in mRNA levels of smooth muscle α actin (*Acta2;* α-SMA), a marker of activated myofibroblasts, and the α1 chain of type 1 collagen (*Col1a1*), one of the fibrotic ECM proteins (Figure 1D), compared with the *SARAWT* mice treated with PBS. In contrast, mRNA levels of these genes were not significantly different between *SARATg* mice treated with bleomycin or PBS. The increase in collagen deposition was further confirmed by Masson’s trichrome staining and by a collagen protein quantification assay on skin sections (Figure 1, E and F).

### Effects of SARA overexpression on pericyte transdifferentiation toward myofibroblasts.

**Figure 2:**
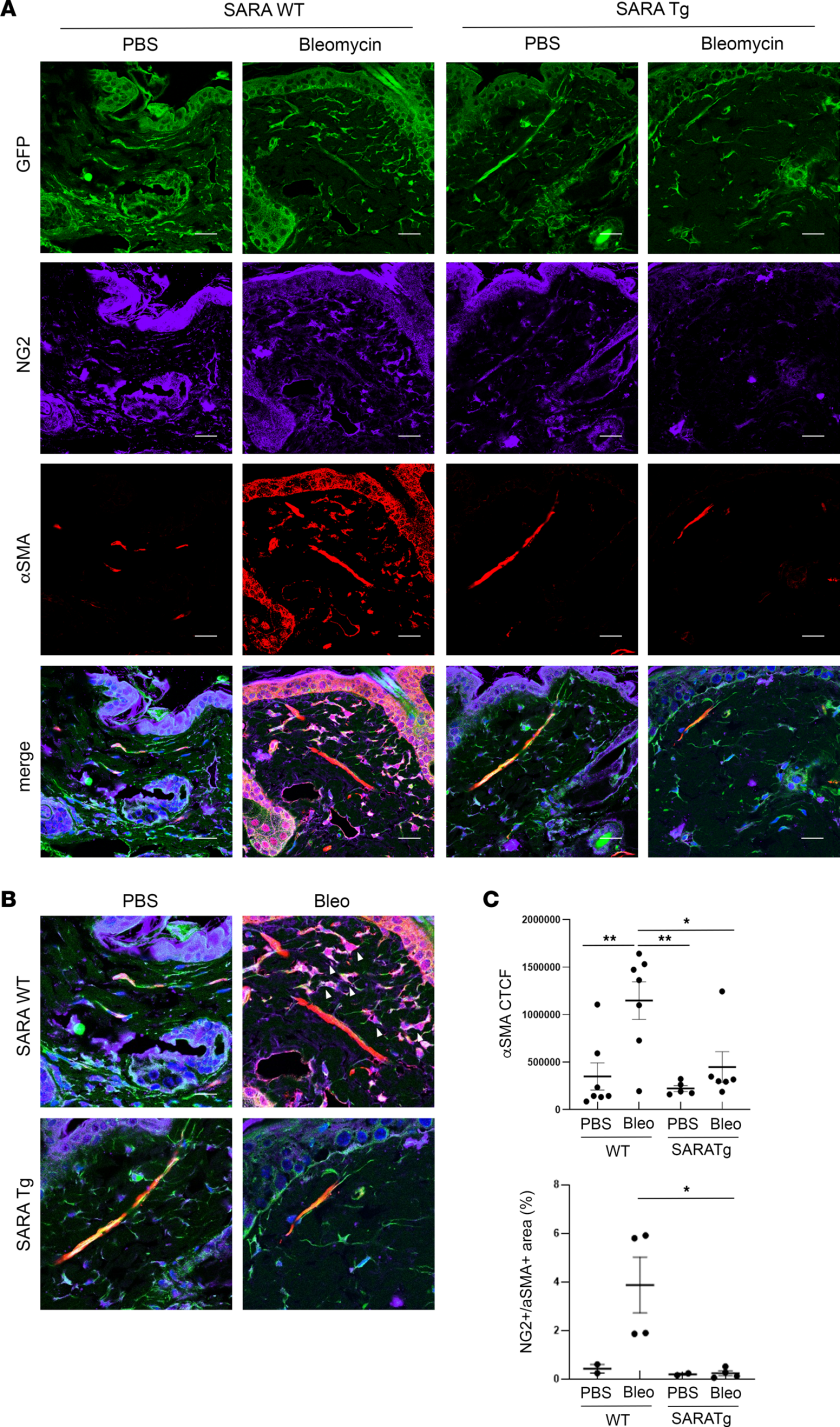
Effects of SARA on pericyte transdifferentiation. Representative images of immunofluorescence staining on skin sections for pericyte marker NG2 (purple) and myofibroblast marker α-SMA (red) are shown (**A**). Pericytes are also expressing GFP in our animal model. Single channels and merged images are shown in the panel. Scale bar = 20 μm. Higher magnification for merged images is shown (**B**). The arrowheads in the merged image indicate the NG2^+^α-SMA^+^ cells in WT bleomycin-treated samples. Scale bar = 10 μm. Representative images from 3 independent experiments are shown. Negative control images are shown in Supplemental Figure 2B. The corrected total cell fluorescence (CTCF) quantification for α-SMA signal and the percentage of double-positive area for NG2^+^ and α-SMA^+^ in dermal layer are shown in the graphs (**C**). One-way ANOVA followed by Tukey’s multiple comparisons test: *P < 0.05, **P < 0.01.

We engineered a mouse model that expresses green fluorescent protein (GFP) in PDGFR-β^+^ cells (*PDGFRb-Cre Z/EG*) (40, 46) to trace the pericytes. In normal skin, α-SMA is expressed in vascular smooth muscle cells and pericytes specifically alongside the arterioles (38). As expected, the pericyte markers NG2 (Figure 2) and CD146 (Supplemental Figure 2) were only detected in a subpopulation of GFP^+^ cells that were localized along blood vessels, marked by α-SMA expression in vascular smooth muscle cells, in *SARAWT* mice treated with PBS (Figure 2A and Supplemental Figure 2A). In *SARAWT* mice treated with bleomycin, an additional population of NG2^+^ (or CD146^+^ in Supplemental Figure 2) cells coexpressed α-SMA, and these cells were different in shape and located in the dermal layer farther away from blood vessels (Figure 2A and Supplemental Figure 2A; arrowheads in Figure 2 indicate the NG2 and α-SMA double-positive cells), suggesting that these pericytes underwent phenotypic switch, expressing α-SMA as a myofibroblast marker. In contrast, in *SARATg* mice, NG2^+^ (or CD146^+^ in Supplemental Figure 2) cells were observed only adjacent to α-SMA^+^ blood vessels, and no additional α-SMA^+^ cells in the dermal layer were observed even after bleomycin treatment (Figure 2, A and B).

Quantification of fluorescence signal of the NG2 and α-SMA double-positive cells in the dermis demonstrated a significant increase in α-SMA signal, more specifically α-SMA and NG2 double-positive cells in *SARAWT* mice treated with bleomycin, but not in *SARATg* mice (see the graphs in Figure 2C). These results suggest that pericytes underwent a phenotypical switch toward myofibroblast-like cells in *SARAWT* mice but not in *SARATg* mice.

**Figure 3:**
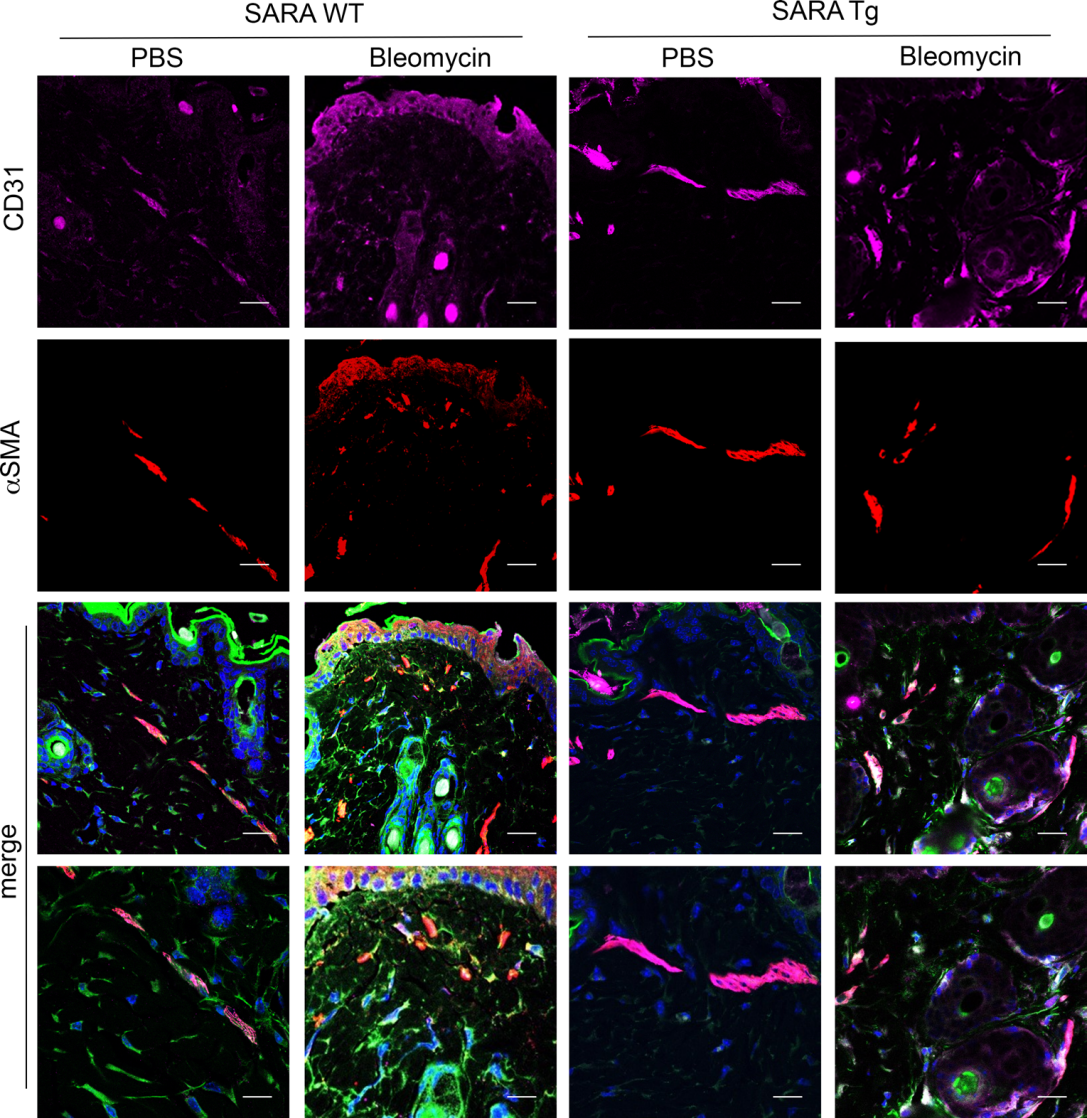
Effects of SARA on α-SMA^+^ cell localization. Representative images of immunofluorescence staining on skin sections for blood vessel marker CD31 (purple) and α-SMA (red) are shown. Merged images are shown in the panel and higher magnification of the latter are provided. Scale bar = 20 μm and 10 μm in the higher magnification. Representative images from 3 independent experiments are shown.

The location of the α-SMA^+^ cells was confirmed by visualizing blood vessels with vascular endothelial cells stained with the endothelial cell marker CD31 (Figure 3). In *SARAWT* mice treated with PBS, α-SMA^+^ cells were observed only in proximity to blood vessels, as expected, suggesting they are either healthy pericytes on arterioles or vascular muscle cells. By contrast, in *SARAWT* mice treated with bleomycin, α-SMA–expressing cells were also observed in the dermal area not physically associated with vascular structures marked by CD31, suggesting that those cells, presumably a subset of NG2^+^ pericytes according to the previous figure, underwent transdifferentiation to myofibroblasts and migrated out from the blood vessels. This change was not observed in *SARATg* mice treated with bleomycin (Figure 3).

**Figure 4:**
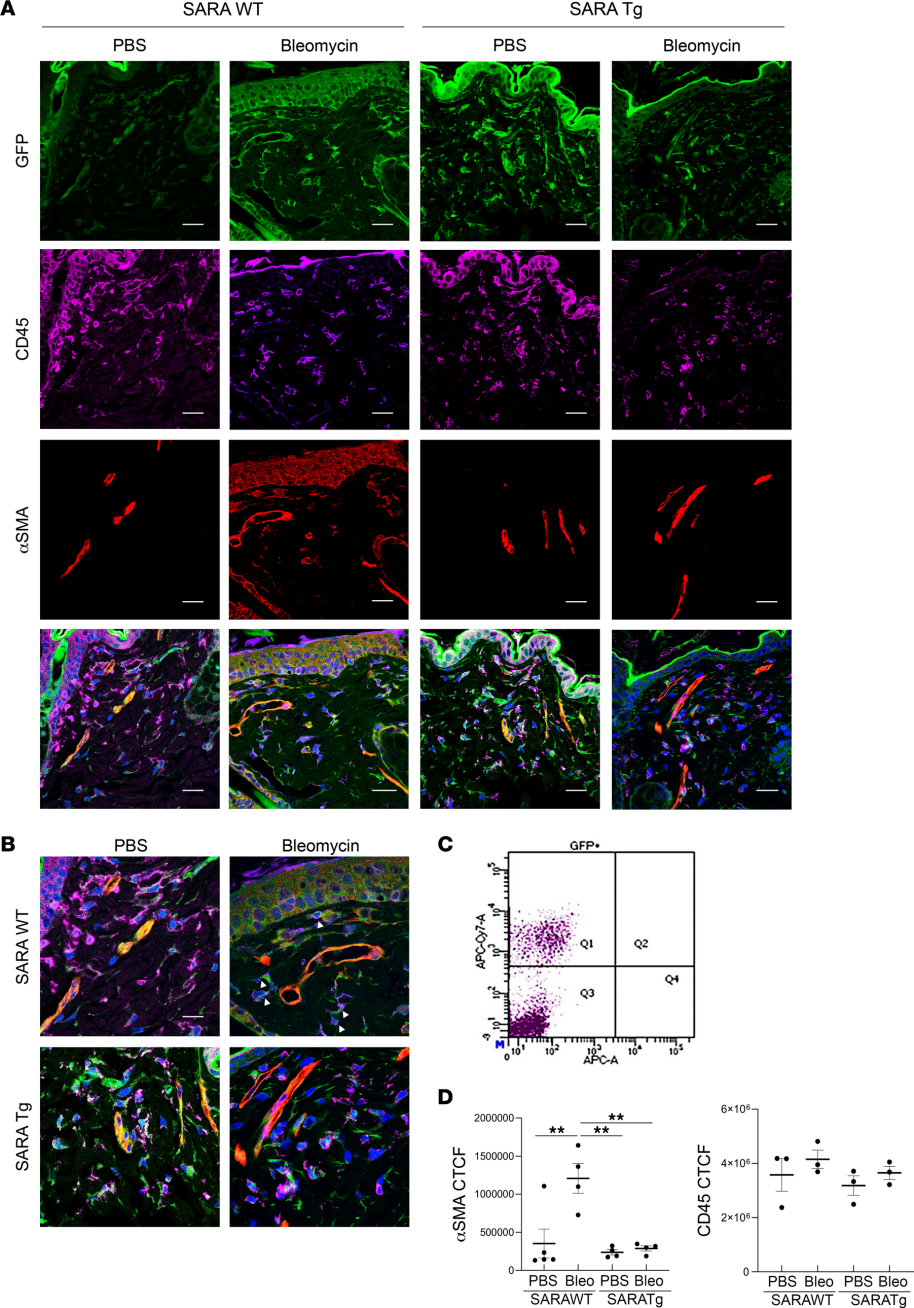
Inflammatory cells detected by PDGFR-β^+^GFP^+^ cells in the *PDGFRb-Cre Z/EG* mice during fibrogenesis. Representative images of immunofluorescence staining on skin sections for inflammatory cell marker CD45 and myofibroblast marker α-SMA are shown (**A**). Single channels and merged images are shown in the panel. Scale bar = 20 μm. Higher magnification for merged images is shown (**B**). The arrowheads in the merged image indicate the CD45**^+^** and α-SMA^–^ cells in the WT bleomycin-treated samples. Scale bar = 10 μm. The scatterplot of the flow cytometric analysis of the PDGFR-β^+^ cells isolated from healthy *SARAWT* or *SARATg* mouse skin and stained for CD45 is shown (**C**). Details for the flow analyses are provided in Supplemental Figure 3. The CTCF quantification for α-SMA and CD45 signal is shown in the graphs (**D**). One-way ANOVA followed by Tukey’s multiple comparisons test: **P < 0.01. Representative images from 3 independent experiments are shown.

During embryogenesis, the PDGFR-β promoter is also active in inflammatory cells (47). Indeed, in *PDGFRb-Cre Z/EG* mice, not all PDGFR-β^+^GFP^+^ cells expressed pericyte markers but did express the pan-leukocyte marker, CD45. Quite a few CD45^+^GFP^+^ cells were observed in the dermis of *PDGFRb-Cre Z/EG* mice treated with bleomycin as well as those treated with PBS (Figure 4, A and B). Flow cytometric analyses of the PDGFR-β^+^ cells isolated from healthy *SARAWT* or *SARATg* mouse skin showed that 30% of them were CD45^+^ cells (Figure 4C and Supplemental Figure 3). The CD45^+^ cell infiltration in healthy skin was expected and presumably due to inflammatory reactions caused by a subcutaneous injection, even with PBS. CD45^+^ cell numbers in the dermis were not significantly different between the *SARAWT* or *SARATg* treated with bleomycin or PBS; on the contrary, α-SMA^+^ cells were significantly increased (graphs in Figure 4D). Importantly, the CD45^+^ cells were negative for α-SMA (arrowheads in Figure 4, A and B, indicate the CD45^+^α-SMA^–^ cells). In *SARATg* mice, we detected similar numbers of CD45^+^ cells as well, and they were not α-SMA^+^. Together, these results suggest that *SARA* overexpression in pericytes prevents their transdifferentiation toward ECM-producing myofibroblasts responsible for the fibrotic phenotype, whereas CD45^+^ cells do not undergo such transdifferentiation.

### Identification of pericyte subpopulations responsible for fibrogenesis and modulated by SARA overexpression.

Our findings support the notion that preventing pericyte to myofibroblast transdifferentiation could be a novel therapeutic approach in SSc and that SARA inhibits the transdifferentiation. Since pericytes are heterogenous and diverse, we aimed to identify specific subpopulations of pericytes that transdifferentiate to myofibroblasts by investigating the molecular profiles of individual cells in fibrosing skin using single-cell RNA-Seq (scRNA-Seq) analysis. To include the majority, if not all, of pericytes, we used PDGFR-β as one of the most commonly expressed pericyte markers. PDGFR-β^+^GFP^+^ cells were isolated from skin of the *PDGFRb-Cre Z/EG*, *SARATg*, or *SARAWT* mice treated with PBS or bleomycin (Supplemental Figure 3) and subjected to single-cell transcriptome analysis. Quality controls of scRNA-Seq analyses are reported in Supplemental Figure 4.

**Figure 5:**
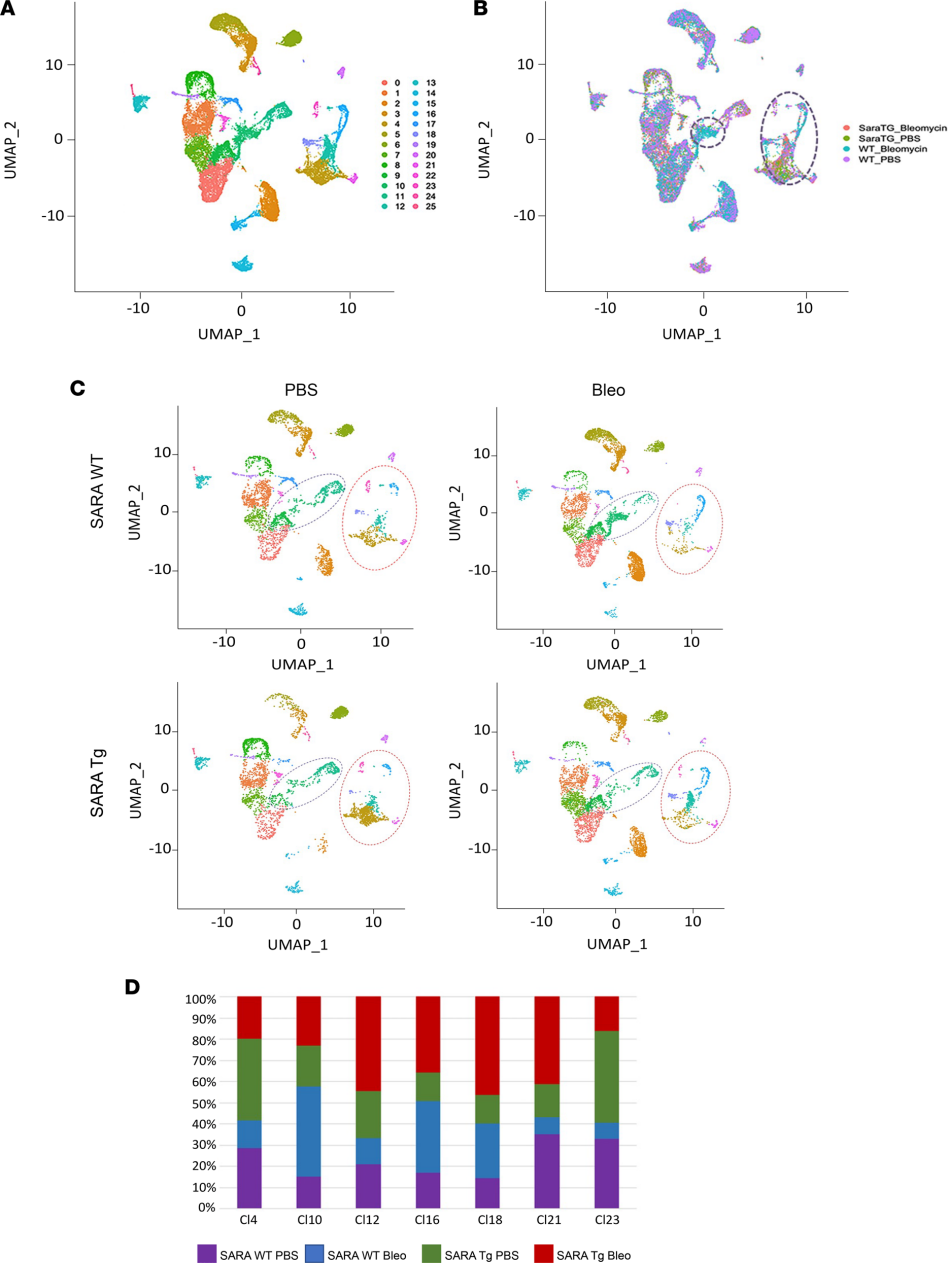
scRNA-Seq analysis of PDGFR-β^+^ cells and subcluster distribution. PDGFR-β^+^ cells isolated from *PDGFRb-Cre/GFP/SARATg* or *SARAWT* mouse skin were sorted by flow cytometry and subjected to scRNA-Seq. Representative UMAP plot of the 25 different clusters revealed by Seurat analysis conducted in R Studio, with all the experimental conditions together, is shown (**A**). Cluster distribution in all 4 experimental conditions together, each represented by different colors in the plot (**B**). Representative UMAP plots for each condition are also shown (**C**). The clusters that changed the most by experimental conditions are circled. The sample component of these clusters in the 4 different experimental conditions is represented in the bar plot in **D**. Representative plots from 3 independent experiments are shown.

Unsupervised uniform manifold approximation and projection (UMAP) clustering performed with Seurat package of the sequencing data revealed 25 distinct clusters (Figure 5A). The clusters were primarily divided in 2 groups, pericytes and inflammatory cells (Figure 5A). In addition, a small cluster of endothelial cells and keratinocytes were identified. Among the clusters, only a few showed a substantial change during fibrogenesis that was reverted with *SARA* overexpression. In more detail, cluster 10 was enriched with cells isolated from *SARAWT* mice treated with bleomycin while clusters 4 and 12 were depleted after bleomycin injection (circled areas in Figure 5B); with *SARA* overexpression these changes were abrogated. These dynamics were more evident when clusters were plotted separately for each condition (Figure 5C). The sample component of these clusters is also shown in the bar graph in Figure 5D.

**Figure 6:**
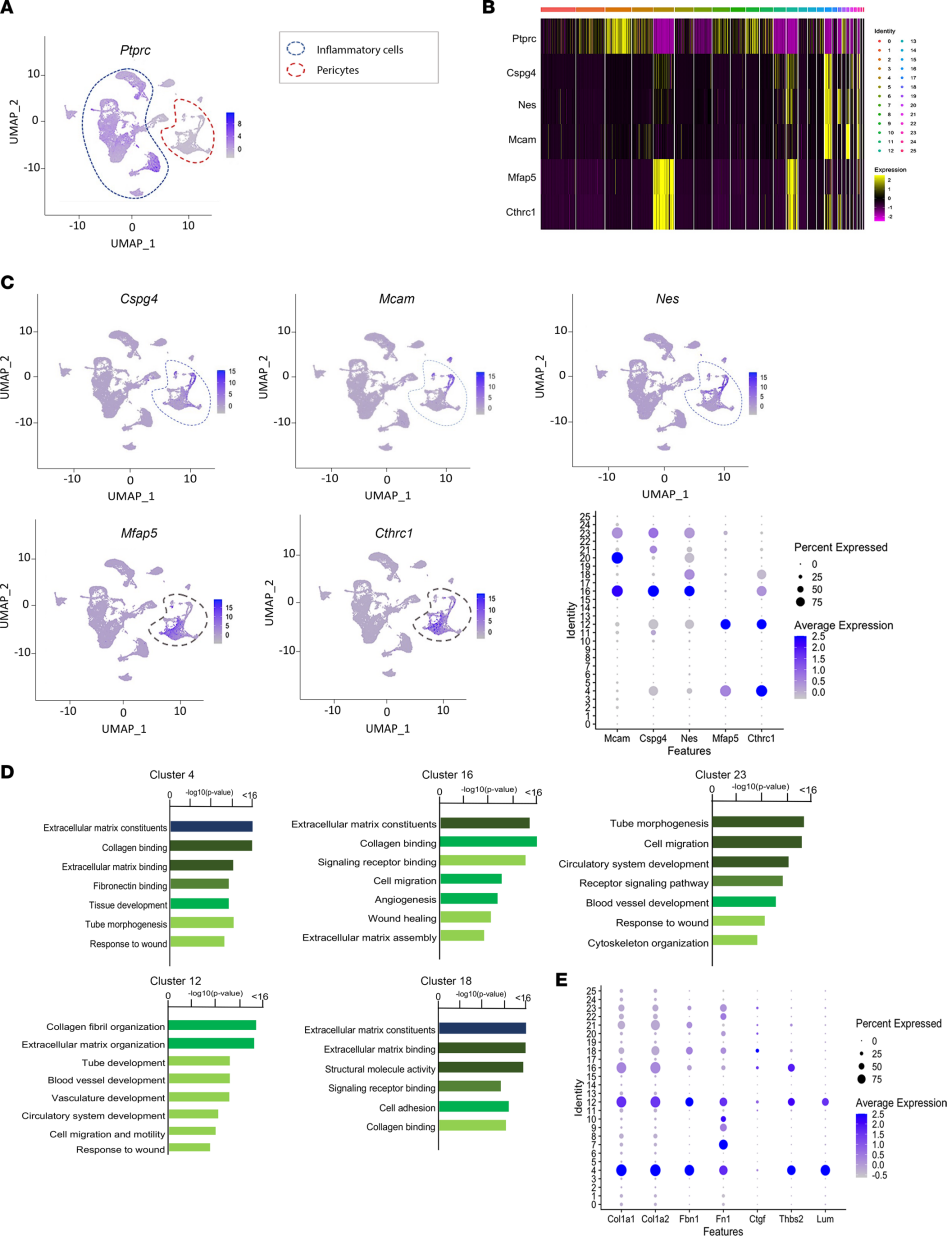
scRNA-Seq analysis on pericyte subclusters. Feature plot and cluster annotation following scRNA-Seq analysis, conducted with Seurat package in R Studio, is shown. Inflammatory cells and noninflammatory cells, annotated as pericytes, were detected based on the expression level of *Ptprc* gene, coding for the pan-leukocyte marker CD45 (**A**). Heatmap reporting the different gene expression profiles of the inflammatory cells and pericytes is shown (**B**). Feature plots and dot plots for pericyte cluster identification, based on the expression of canonical markers *Cspg4* coding for NG2, *Mcam* encoding for CD146, *Nestin*, and noncanonical markers *Mfap5* and *Cthrc1*, are shown (**C**). gProfiler pathway enrichment analysis based on the differentially expressed genes of the pericyte clusters 4, 12, 16, 18, and 23, and dot plot of profibrotic gene expression, *Col1a1*, Col1*a*2, *Fn1*, *Fbn1*, *Ctgf*, *Thbs2*, and *Lum*, are shown (**D** and **E**). Representative plots from 3 independent experiments are shown.

Consistent with our immunofluorescence evaluation, the clusters were largely divided into 2 large populations, pericytes and inflammatory cells (Figure 6A). A total of 6 clusters (clusters 4, 12, 16, 18, 21, 23; Figure 6A, circled by red line) were annotated as pericytes. A heatmap revealed that they share common markers, and those markers are not expressed by the *Ptprc*-expressing (coding for CD45) populations (Figure 6B). As shown in the feature plots and dot plots, some of the pericyte clusters (clusters 16, 23) expressed the canonical markers NG2 (encoded by *Cspg4* gene), CD146 (encoded by *Mcam* gene), and nestin (encoded by *Nes* gene), whereas other clusters (clusters 4 and 12) in the vicinity of canonical pericyte clusters did not express these canonical markers (Figure 6C) and for this reason were annotated as “noncanonical” pericytes. The cells in noncanonical pericyte clusters 4 and 12 expressed *Mfap5* (microfibril associated protein 5), which is involved in maintenance of vessel integrity (48), and *Cthrc1* (collagen triple helix repeat containing 1), which is involved in vascular remodeling (49), indicating that these clusters are physiologically related to pericytes. As mentioned above, within the pericyte populations, clusters 4 and 12 diminished after bleomycin treatment in *SARAWT* mice, while they were preserved in *SARATg* mice (Figure 5C).

gProfiler analysis of differentially expressed genes in all pericyte clusters revealed enrichment in pathways involved in vasculature development and morphogenesis, further supporting the notion that they are pericytes. In addition, genes associated with ECM assembly and organization were also enriched in some of the pericyte clusters (Figure 6D). Importantly, the expression of the profibrotic markers *Col1a1*, *Col1a2*, fibronectin 1 (*Fn1*), fibrillin-1 (*Fbn1*), connective tissue growth factor (*Ctgf*), thrombospondin 2 (*Thbs2*), and lumican (*Lum*) was seen mostly in the clusters of pericytes that do not express canonical pericyte markers (Figure 6E), suggesting that this subset of pericytes specifically assumes a myofibroblast-like phenotype.

**Figure 7:**
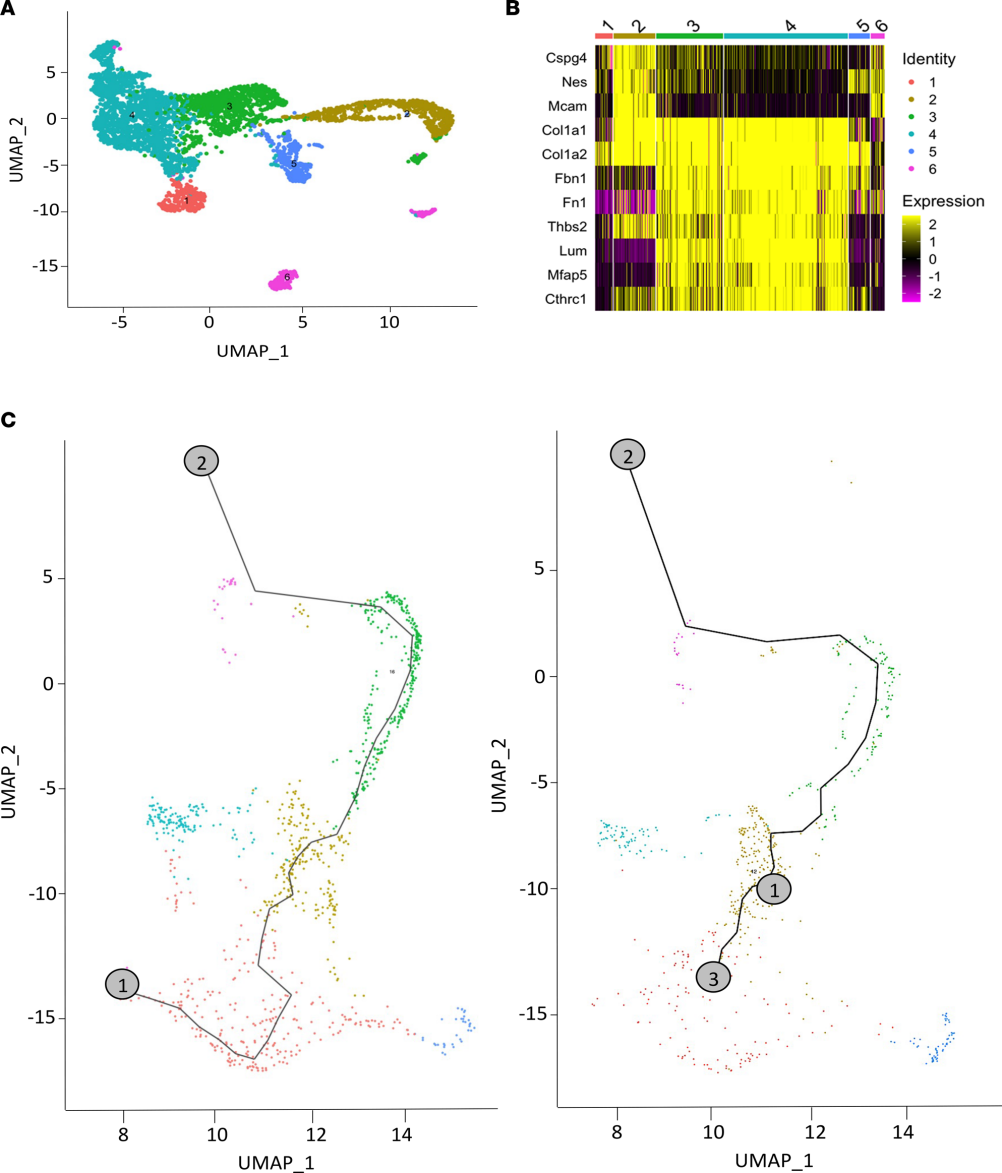
Trajectory inference analyses on pericyte subclusters. Reclustering of pericyte clusters (clusters 4, 12, 16, 18, 21, 23 in the original analyses) is shown (**A**). Heatmaps of the canonical markers and profibrotic markers in the pericyte clusters are shown (**B**). Trajectory inference analysis of the pericyte clusters between *SARAWT* and *SARATg* upon bleomycin treatment is shown (**C**). Representative plots from 3 independent experiments are shown.

Seurat reclustering of the 6 clusters identified as pericytes is shown in Figure 7A. The heatmap in Figure 7B clearly shows that the profibrotic genes were mainly expressed in the noncanonical pericyte populations, even if their expression was detected in the canonical pericytes at a lower level, confirming the evidence that a fraction of pericytes is more prone to transdifferentiate to myofibroblasts during fibrogenesis.

Trajectory analysis of the pericyte clusters only confirmed that within *SARAWT* mice, the pericytes had a single-direction trajectory between the noncanonical pericyte cluster 4 and canonical cluster 6 (cluster 23 in the original plot) (Figure 7C). As previously mentioned, cluster 3 (cluster 12 in original analysis) was devoid in bleomycin-treated *SARAWT* mice, so there was this single directionality toward the canonical pericytes and an increase of them, probably due to a need to replace physiological pericytes in the tissue. In contrast, in *SARATg* samples, there was a branching point in cluster 3, which was preserved, that created an opposite, but truncated, direction toward cluster 4. These results suggest that *SARA* overexpression regulates the dynamics of the cells, specifically in the noncanonical pericyte clusters, preventing the fibrogenic path observed in the *SARAWT* mice.

Differentially expressed genes in the cluster located at the branching point of trajectory, cluster 3, play a critical role in determining the cellular fate. As shown in Table 1, among the top 5 significantly downregulated genes in *SARATg* samples, there are *Nme2* and *Gas5*, and among the top 5 upregulated genes with *SARA* overexpression, there is *Cyr61*. NME2 is a histidine kinase involved in TGF-β–induced activation of hepatic stellate cells, a liver pericyte prototype, and CCl4-induced liver fibrosis (50), and *Gas5* is a long noncoding RNA whose high levels are associated with liver fibrosis (51). In contrast, CYR61 attenuates the TGF-β signaling–mediated fibrosis (52), and it was described as an antifibrotic mediator in SSc (53).

**Figure 8:**
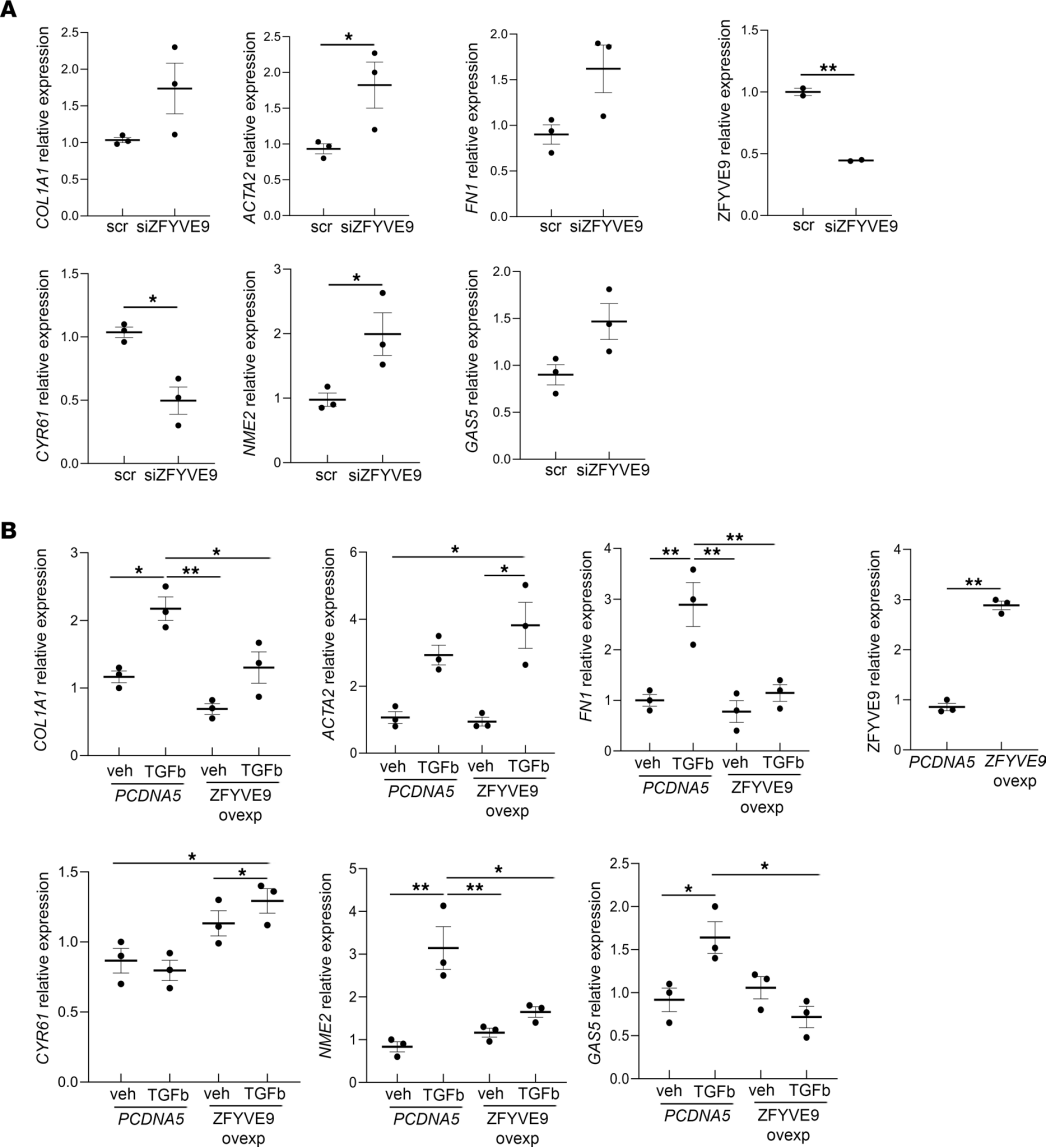
Effect of *SARA* downregulation and overexpression on human pericytes’ transdifferentiation. mRNA expression for profibrotic genes *COL1a1*, *ACTA2*, *FN1*, *NME2*, and *GAS5* and for antifibrotic gene *CYR61*, after downregulation of endogenous SARA (*ZFYVE9*) levels by siRNA assay, is shown (**A**). Summary of 3 independent experiments is shown. Mann-Whitney test: *P < 0.05, **P < 0.01. mRNA expression for profibrotic genes *COL1a1*, *ACTA2*, *FN1*, *NME2*, and *GAS5* and for antifibrotic gene *CYR61*, after overexpression of SARA (*ZFYVE9*) and upon 24-hour TGF-β treatment is shown (**B**). Summary of 3 independent experiments is shown. One-way ANOVA followed by Holm-Šídák multiple comparisons test: *P < 0.05, **P < 0.01. *ZFYVE9* gene expression downregulation and overexpression are shown in the figure. scr, scrambled siRNA; *PCDNA5*, TGF-β1 plasmid.

In vitro experiments were performed to verify the role of SARA in pericyte transdifferentiation. SARA downregulation by siRNA interference in cultured human pericytes (si*ZFYVE9*) showed a substantial upregulation of profibrogenic genes such as *COL1a1*, *ACTA2*, and *FN1*. Moreover, we observed a modulation of the genes that were identified in our scRNA-Seq analysis, specifically in cluster 3 (cluster 12 in the original plot), where the critical branching point was observed. The antifibrotic gene *CYR61* was downregulated, and the profibrotic genes *NME2* and *GAS5* were upregulated (Figure 8A) by SARA silencing. Contrariwise, SARA overexpression in cultured pericytes (*ZFYVE9* overexpressing) attenuated upregulated expression of *COL1a1*, *ACTA2*, and *FN1* as well as *NME2* and *GAS5* by TGF-β1. TGF-β1 treatment also slightly increased *CYR61* expression. SARA overexpression also significantly downregulated expression *CYR61* at baseline and after TGF-β1 treatment (Figure 8B). These findings imply that SARA prevents pericyte transition toward myofibroblast characteristics during fibrosis via modulating these molecules.

### Colocalization of noncanonical pericyte markers and α-SMA in fibrotic mouse skin and in SSc patient skin.

**Figure 9:**
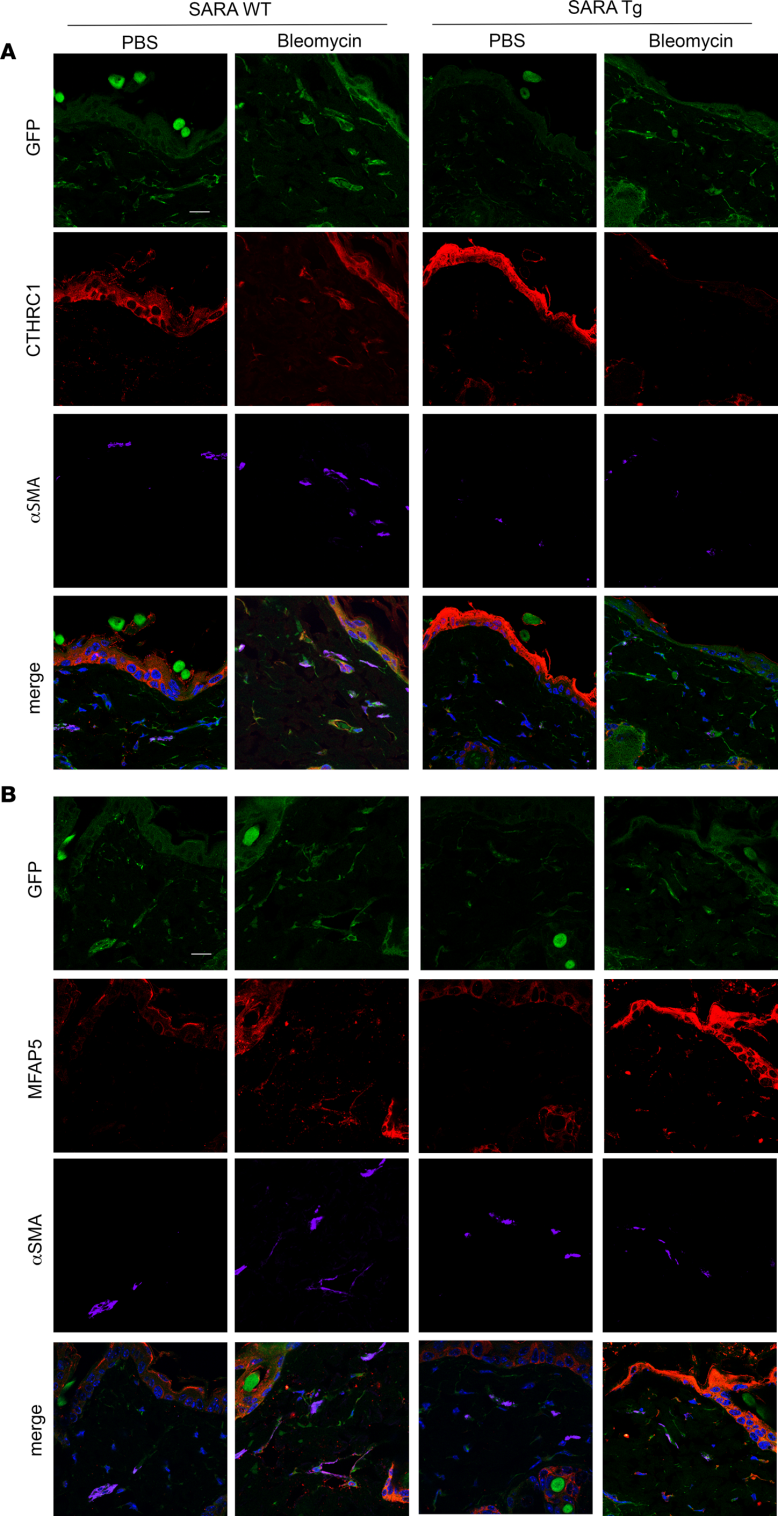
Effect of SARA on noncanonical pericyte transdifferentiation. Representative images of immunofluorescence staining on skin sections for noncanonical pericyte markers CTHRC1 (**A**) and MFAP5 (**B**) and myofibroblast marker α-SMA are shown. The arrowheads in the merged image indicate the CTHRC1/α-SMA^+^ and the MFAP5/α-SMA^+^ cells in the WT bleomycin-treated samples. Single channels and merged images are shown in the panel. Scale bar = 10 μm. Representative images from 3 independent experiments are shown. Negative control images are shown in Supplemental Figure 2B.

**Figure 10:**
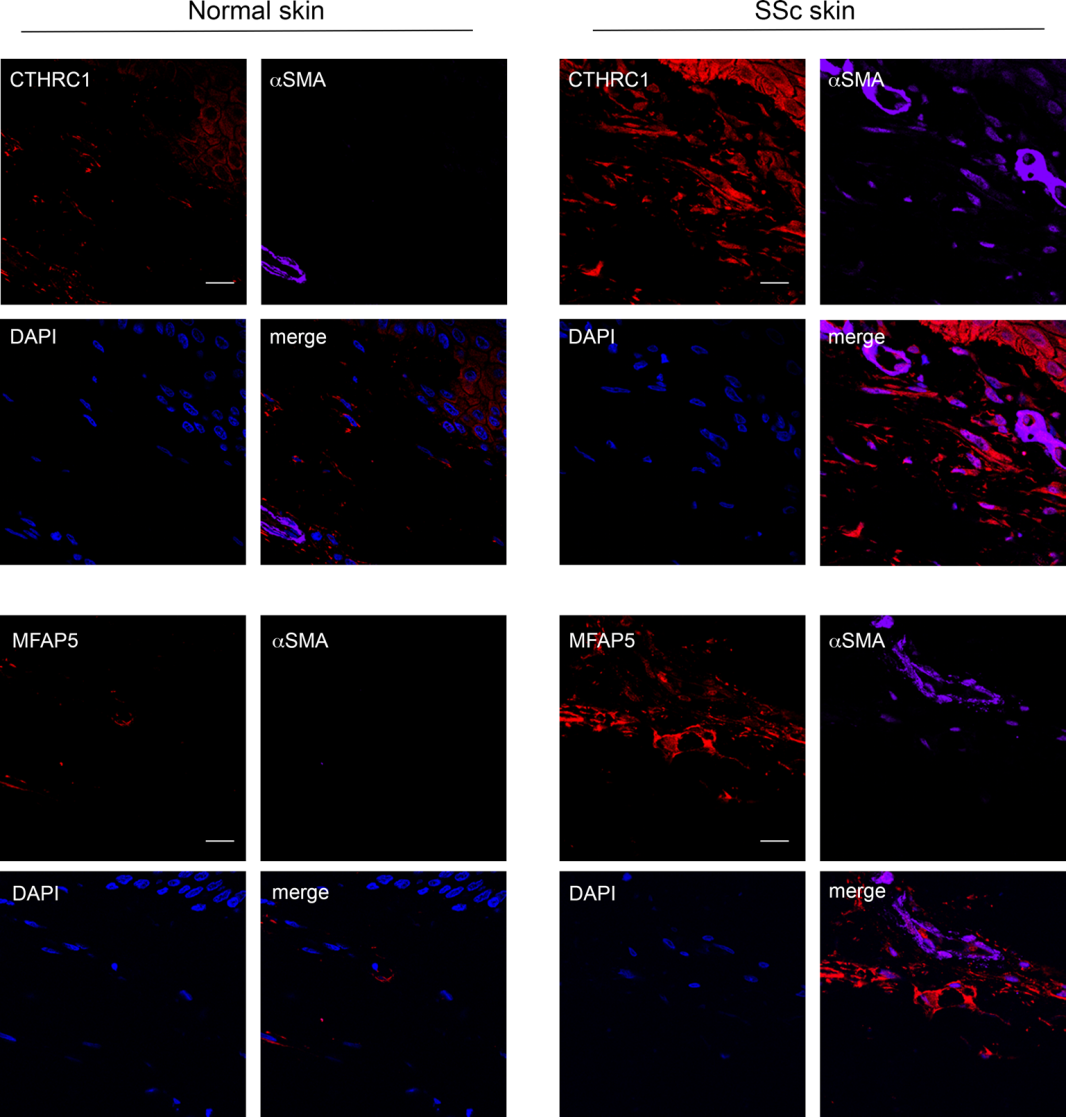
Colocalization of noncanonical pericyte markers and α-SMA in SSc patient skin. Representative images of immunofluorescence staining on normal (left) and SSc patient (right) skin sections for noncanonical pericyte marker CTHRC1 (red) and MFAP5 (red) and myofibroblast marker α-SMA (purple) are shown. Single channels and merged images are shown in the panel. Scale bar = 10 μm. Representative images from 3 independent experiments are shown. Negative control images are shown in Supplemental Figure 2B.

To further investigate the role for the noncanonical pericyte markers identified in our scRNA-Seq analysis, we reanalyzed the mouse skin sections and performed double immunostaining for those markers and α-SMA. As shown in Figure 9, both CTHRC1 (Figure 9A) and MFAP5 (Figure 9B) were identified among the GFP^+^ cells in our animal model, and they both colocalized with α-SMA in *SARAWT* mouse skin treated with bleomycin, underlining that CTHRC1- and MFAP5-expressing cells are more prone to transdifferentiate toward myofibroblast lineage and are activated in fibrogenesis. The colocalization was not observed in healthy skin, and importantly, not in skin of the *SARATg* treated with bleomycin. To unravel the clinical relevance of those subsets of pericytes, we performed immunostaining for CTHRC1 and MFAP5 using SSc patient skin (Figure 10). Similar to the mouse skin, the colocalization of α-SMA with either CTHRC1 and MFAP5 was clearly observed in SSc patients but not in healthy skin tissue. In addition, scRNA-Seq of SSc patient skin demonstrated the expression of these genes in myofibroblast-like cells (J Varga and JE Gudjonsson at University of Michigan, unpublished observations).

### Th2 cell and macrophage activation in fibrosis and role for SARA.

**Figure 11:**
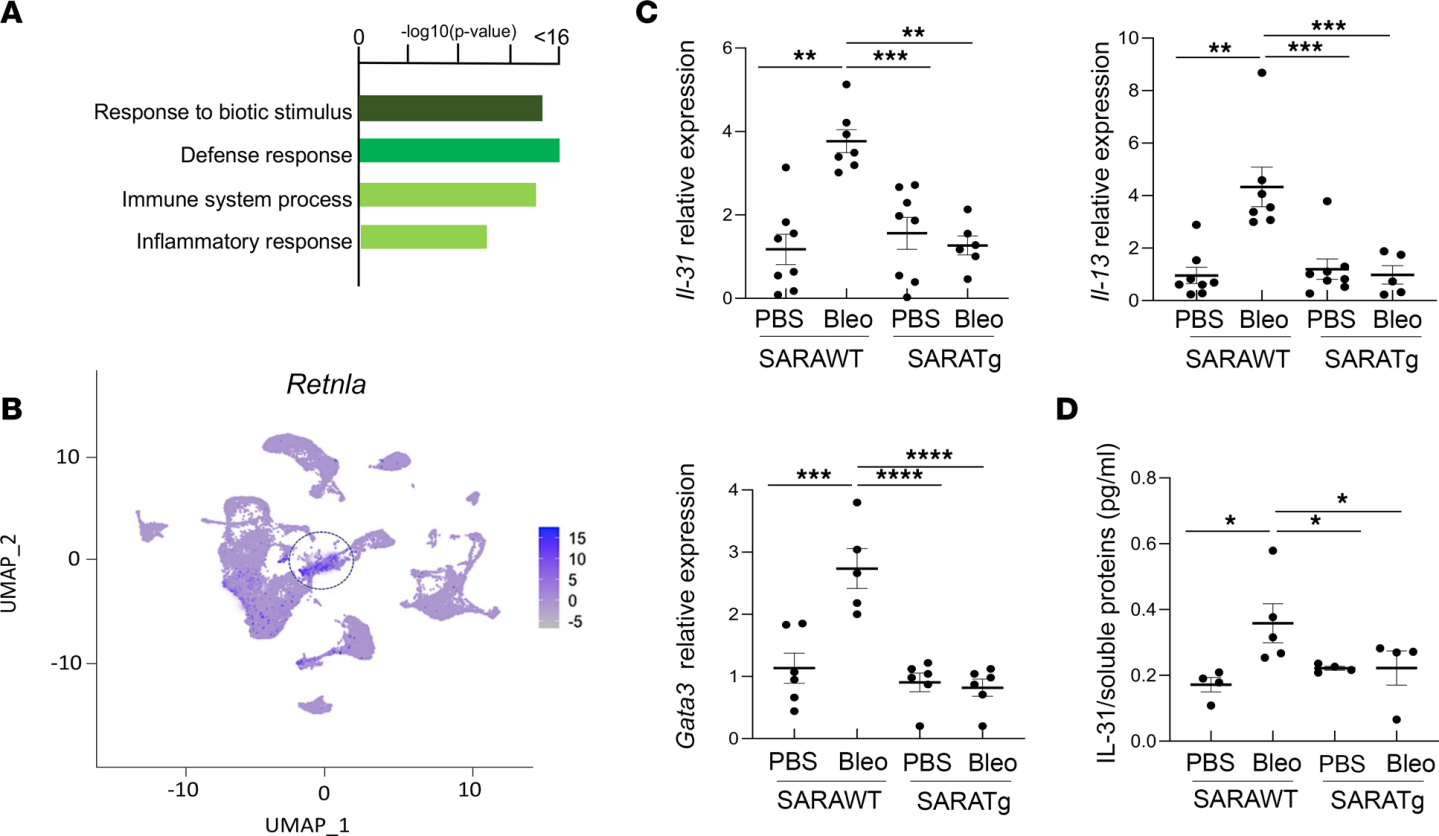
Th2 cell activation during fibrogenesis. gProfiler pathway enrichment analysis of cluster 10 is shown in **A**. Cluster 10, circled in the plot and enriched in cells in fibrotic animals, had the highest expression of *Retnla*, a downstream effector of IL-31 pathway, a well-known polarized Th2 cytokine (**B**). mRNA expression for *Il-31*, *Il-13*, and *Gata3* (**C**) and protein expression of IL-31 (**D**) evaluated by quantitative PCR and ELISA, respectively, are shown. Each dot represents the expression level from a different mouse. *SARAWT* mice *n* = 15 (PBS treated *n* = 8 and bleomycin treated *n* = 7) versus *SARATg* mice *n* = 15 (PBS treated *n* = 8 and bleomycin treated *n* = 6). One-way ANOVA followed by Holm-Šídák multiple comparisons test: *P < 0.05, **P < 0.01, ***P < 0.001, and ****P < 0.0001.

**Figure 12:**
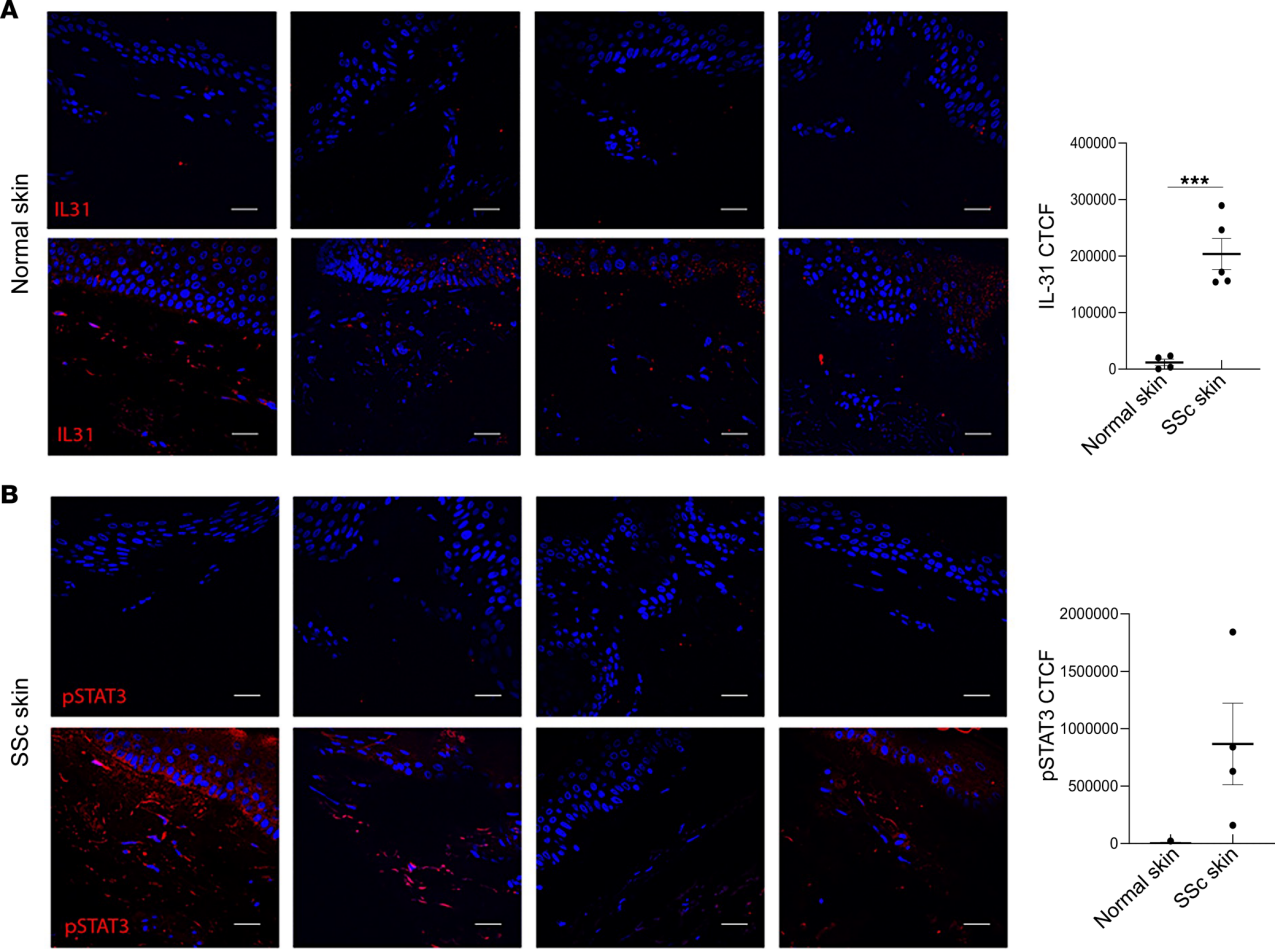
Th2 cell activation in SSc patient skin. Representative images of IL-31 (**A**) and p-STAT3 (**B**) in 4 different SSc skin biopsies, compared with normal skin, are shown. The CTCF quantification for IL-31 and p-STAT3 is shown in the graph. Nuclei were detected with DAPI and the merged snapshots are shown in the panel. Scale bar = 20 μm. SSc skin patients *n* = 4, healthy volunteer skin *n* = 4. Mann-Whitney test: ***P < 0.001.

The other 19 clusters among the PDGFR-β^+^GFP^+^ cell population were represented by inflammatory cells that expressed *Ptprc*, which encodes the pan-leukocyte marker CD45 (Figure 6A, circled by blue line). Inflammation is one of the main manifestations of all autoimmune diseases. Skin from patients with SSc shows inflammatory infiltrates consisting of macrophages, T lymphocytes, and dendritic cells as the predominant cell types (54). Immune cells in skin can also be fibrogenic in SSc (55–59). However, the contributions of each cell type and the chemokines they produce in the profibrotic microenvironment are not well defined. scRNA-Seq analysis of our mouse model skin revealed characteristics of the CD45^+^ cells in fibrosing skin. Specifically, cluster 10 was enriched with cells derived from *SARAWT* mice treated with bleomycin but depleted in skin of the *SARA*-overexpressing mice treated with bleomycin (Figure 5C). This cluster showed enrichment in pathways involved in immune reactions, cytokine and chemokine production, and inflammation (Figure 11A), and one of the most highly upregulated genes was resistin-like molecule α (*Retnla*), a downstream target of Th2 cell–produced cytokines, including IL-31 (Figure 11B). *Retnla* is a regulator of Th2 driven inflammation, and it also regulates M1-M2 switch of macrophages and their activation (60, 61). IL-31 is a cytokine primarily produced by activated Th2 lymphocytes. In fact, mRNA expression levels of cytokines produced by Th2 lymphocytes, *Il-31* and *Il-13*, and the transcription factor involved in Th2 activation, *Gata3*, were significantly increased in *SARAWT* mouse skin, but not in *SARA*-overexpressing mouse skin, after bleomycin treatment (Figure 11C). IL-31 protein levels measured in skin tissue homogenates by ELISA were also increased in *SARAWT* samples, but not in *SARATg* samples, after bleomycin treatment (Figure 11D). We then examined Th2 cell activation in skin biopsy sections from diffuse cutaneous SSc patients and from healthy volunteers (demographics of the samples are reported in Supplemental Figure 5A). As expected, normal skin tissue did not show any signal for IL-31 or phosphorylated STAT3 (p-STAT3), a well-known downstream mediator for IL-31 (62–64). In contrast, SSc skin sections were positive for both IL-31 and p-STAT3. Variability in staining intensity was detected, reflecting the phenotypic heterogeneity of patients with SSc (Figure 12).

In accordance with our hypothesis, SARA was abundantly expressed in normal skin but decreased specifically in the dermis layer of SSc skin (Supplemental Figure 5B). We also evaluated published data sets (GSE9285) analyzing gene expression profiles in SSc skin by Gene Array (33) and found that *SARA* mRNA (*ZFYVE9*) levels were significantly lower in patients with SSc (Supplemental Figure 5C).

Moreover, scRNA-Seq analysis on SSc patients showed that SARA expression was relatively diffused, and it was decreased in SSc patients when compared with healthy volunteers (J Varga and JE Gudjonsson at University of Michigan, unpublished observations).

### A potential crosstalk between the pericytes and immune cells in fibrogenesis.

**Figure 13:**
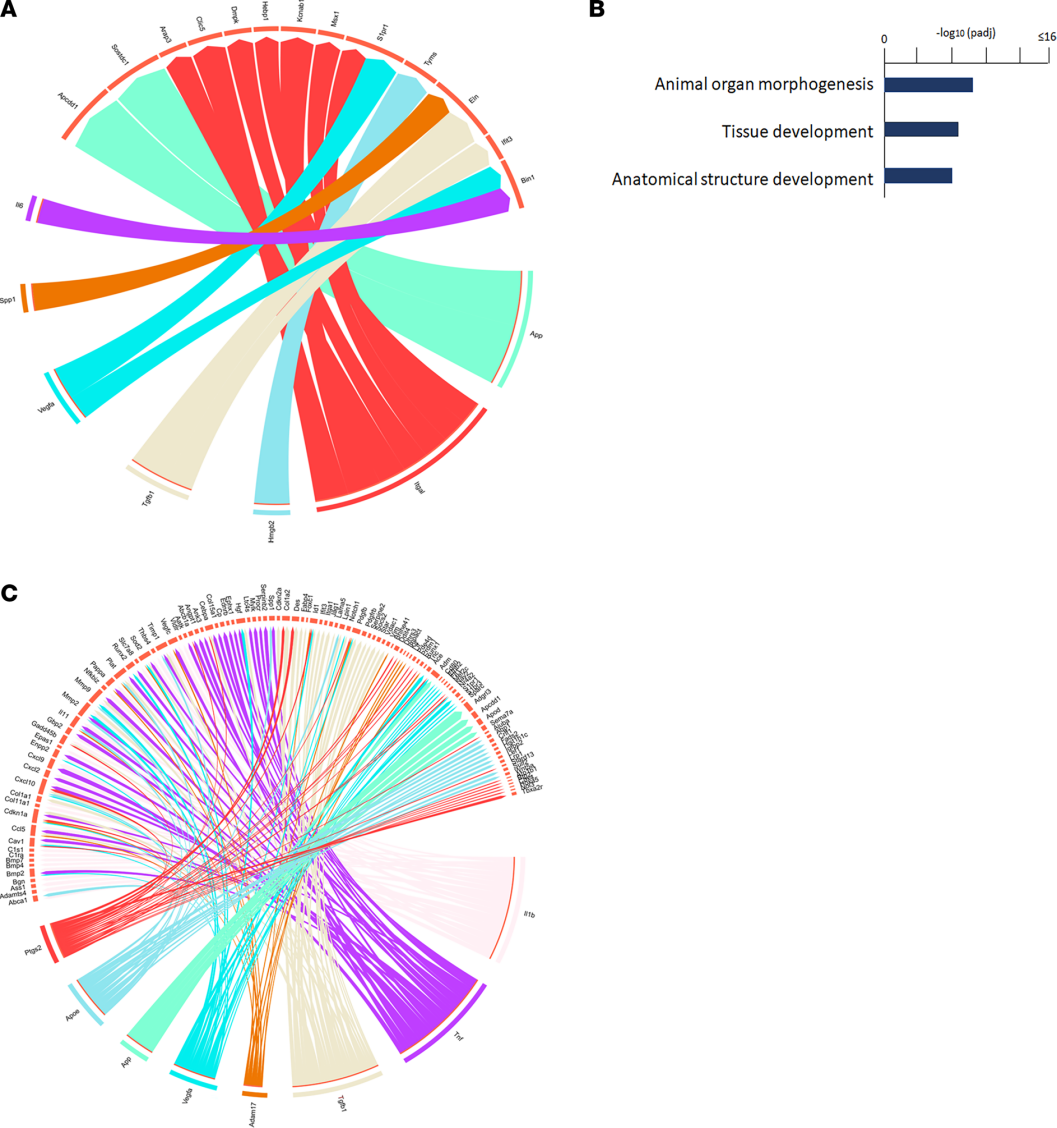
Crosstalk between pericytes and immune cells. Ligand-receptor *NicheNet* analysis in *SARAWT* bleomycin versus *SARAWT* PBS is shown. Ligands from cluster 10 are represented on the bottom of the plot, and target genes in cluster 12 (original analysis) are represented on the top of the plot (**A**). gProfiler pathway analysis of the target gene expression in cluster 12 is shown (**B**). Circos plot of the interaction between cluster 10 (sender) and cluster 12 (receiver) based on ligand-receptor *NicheNet* analysis in *SARATg* bleomycin versus *SARATg* PBS is shown. Ligands from cluster 10 are represented on the bottom of the plot, and target genes in cluster 12 are represented on the top of the plot (**C**). Representative plots from 3 independent experiments are shown.

Activated lymphocytes may also regulate other immune cell behavior and pericyte transdifferentiation through the chemokines they produce in SSc (55). Indeed, our single-cell RNA-Seq data suggested a potential crosstalk between immune cells and pericytes during fibrogenesis. In *SARAWT* mice treated with bleomycin, Th2 lymphocytes were activated and induced macrophage polarization, as shown by *Retnla* expression in cluster 10. In addition, ligand-receptor analysis showed a strong interaction between cluster 10 and 12 subsets of pericytes in *SARAWT* mice (Figure 13A). Among the ligands produced by cluster 10, IL-6 and TGF-β1 were found to significantly drive gene expression in the receiver cluster 12. In the receiver cluster 12, genes related to morphogenesis were associated with the ligand produced by cluster 10 (Figure 13B), in addition to the receptors of IL-31, IL-6, IL-11, and TGF-β (Table 2). IL-6 is a cytokine mainly produced by macrophages (65), and together with IL-31 and IL-11, it has been shown to be involved in skin fibrosis and cutaneous wound healing (66), as well as in SSc progression (67). These data show that the chemokines produced by cluster 10 might act directly on cluster 12, inducing the molecular changes and the transdifferentiation of the subset of pericytes toward a myofibroblast phenotype, as shown by the upregulation of *Col1a1* and *Col1a2* gene expression in cluster 12 (Table 2). In contrast, in *SARATg* mice, many more interactions between clusters 10 and 12 were detected (Figure 13C). Many target genes detected in cluster 12 are encoding ECM proteins, and their expression levels were significantly decreased in *SARATg* mice even after bleomycin treatment (Table 3), while significant interactions with the genes associated with morphogenesis detected in *SARAWT* mice were not detected in *SARATg* mice.

**Figure 14:**
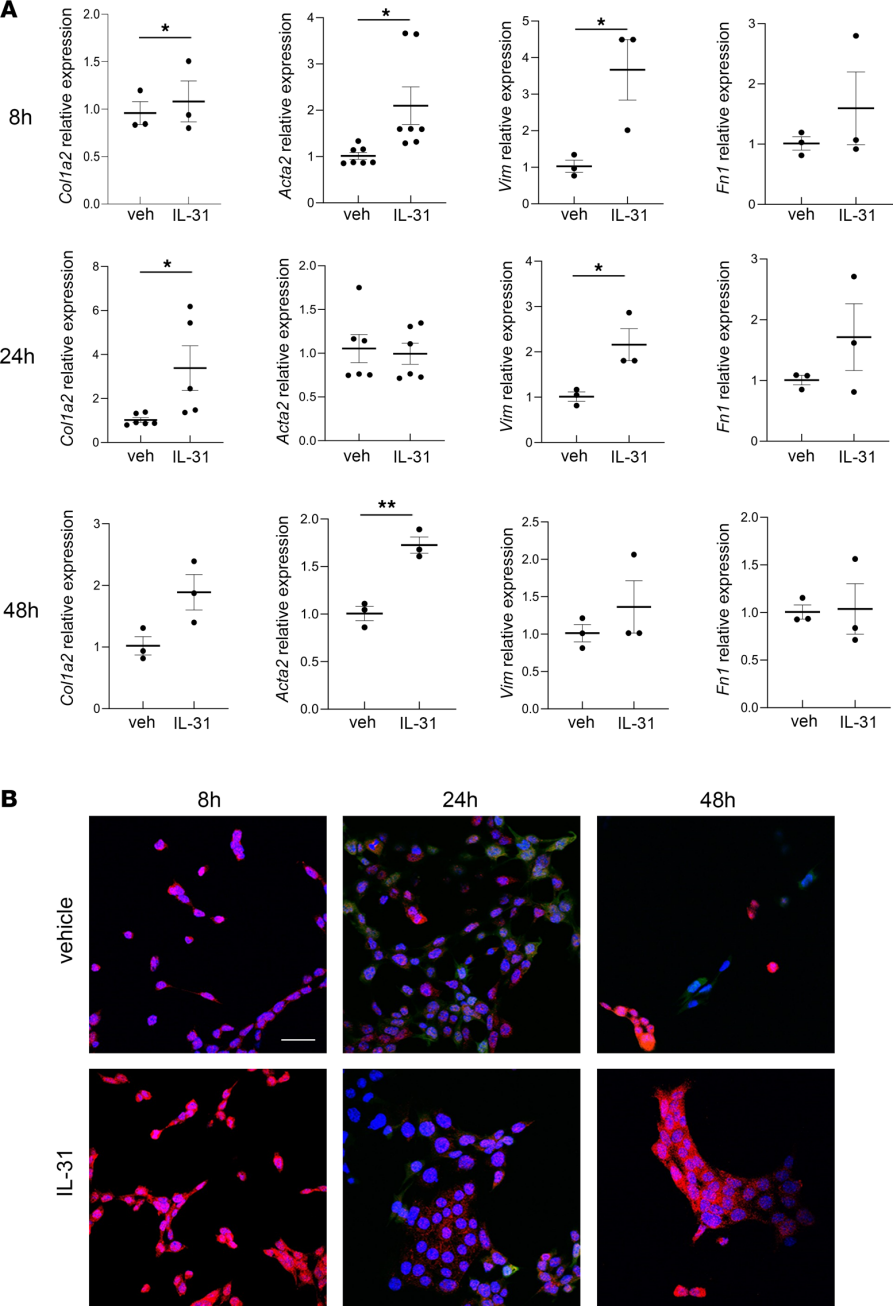
Effects of IL-31 treatment on mouse pericytes. mRNA expression for profibrotic genes *Col1a1*, *Acta2*, *Vim*, and *Fn* after 8 hours, 24 hours, and 48 hours of 50 ng/mL IL-31 treatment, are shown (**A**). Mann-Whitney test: *P < 0.05, **P < 0.01. Representative images of immunofluorescence staining on mouse pericytes for α-SMA after 8 hours, 24 hours, and 48 hours of 50 ng/mL IL-31 stimulation are shown (**B**). Nuclei were detected with DAPI and the merged snapshots are shown in the panel. Scale bar = 20 μm. Representative image and summary from 3 independent experiments are shown.

**Figure 15:**
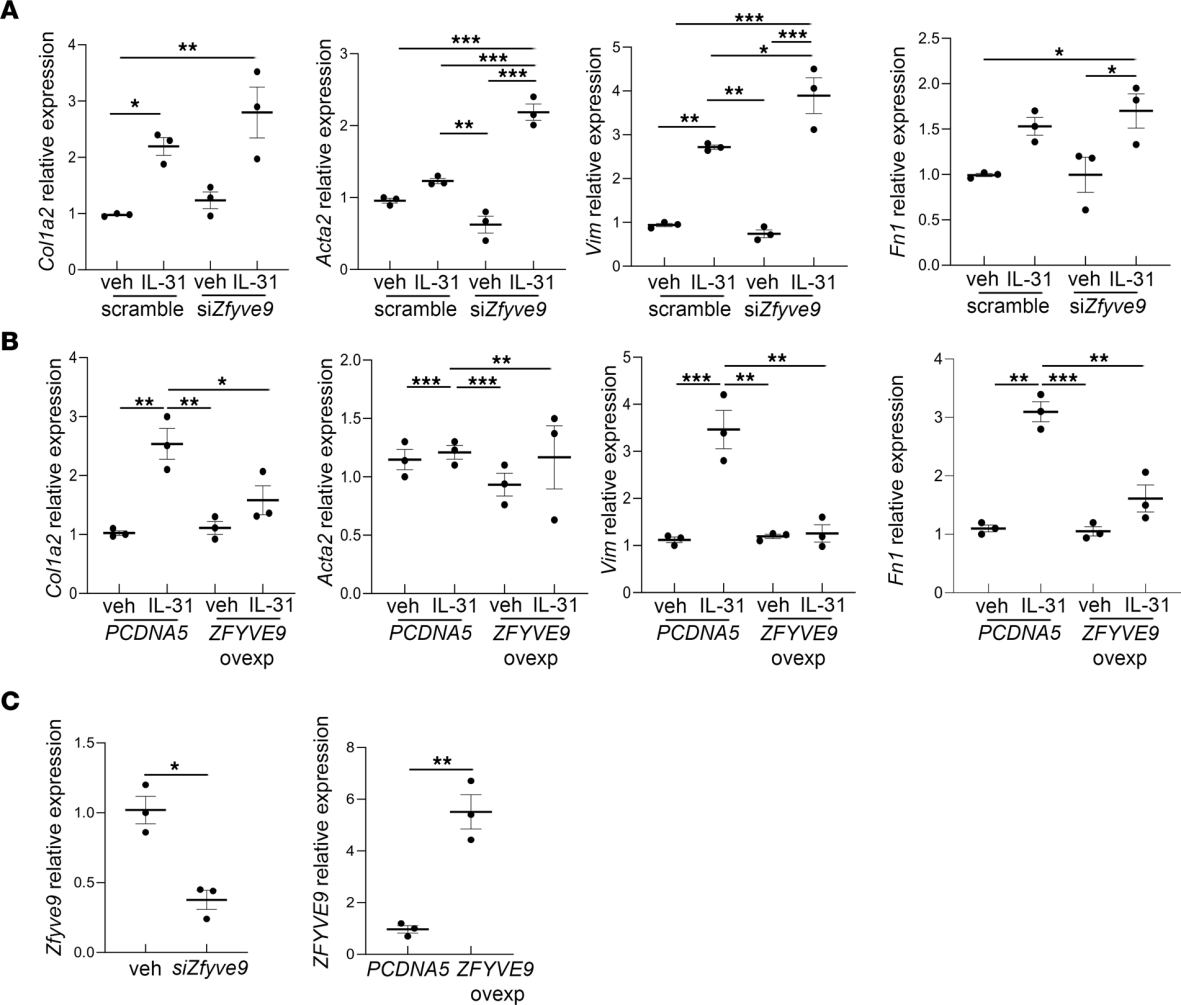
Effect of *SARA* downregulation and overexpression on IL-31 activity in mouse pericytes’ transdifferentiation. mRNA expression for profibrotic genes *Col1a1*, *Acta2*, *Vim*, and *Fn1*, after downregulation of endogenous SARA (*Zfyve9*) levels by siRNA assay and after 24-hour IL-31 stimulation, is shown (**A**). Summary of 3 independent experiments is shown. One-way ANOVA followed by Holm-Šídák multiple comparisons test: *P < 0.05, **P < 0.01, and ***P < 0.001. mRNA expression for profibrotic genes *Col1a1*, *Acta2*, *Vim*, and *Fn1*, after overexpression of SARA (*ZFYVE9*) and upon 24-hour IL-31 treatment, is shown (**B**). *Zfyve9* gene expression downregulation and *ZFYVE9* overexpression are shown (**C**). Summary of 3 independent experiments is shown. One-way ANOVA followed by Holm-Šídák multiple comparisons test: *P < 0.05, **P < 0.01, and ***P < 0.001.

The crosstalk between lymphocytes and pericytes was further evaluated in vitro. Immortalized mouse pericytes treated with IL-31 showed a significant increase in the expression of the profibrotic markers *Col1a1*, *Acta2*, and vimentin (*Vim*). A positive trend for *Fn1* was observed after 8 hours of the treatment, and it remained substantially higher compared with the vehicle-treated cells after 24 hours of stimulation. After 48 hours, *Acta2* expression level was also increased. These results were confirmed by immunofluorescence for α-SMA (Figure 14). To better investigate the involvement of SARA in regulating IL-31 profibrotic activity on pericytes, mouse pericytes were transfected with si*Zfyve9* to downregulate endogenous SARA expression level, and then they were treated with IL-31 for 24 hours. As shown in Figure 15A, with SARA downregulation we observed a strong induction in *Col1a1*, *Fn1*, *Vim*, and *Acta2* expression, which was not observed with IL-31 treatment only. In contrast, when we overexpressed SARA in mouse pericytes (*ZFYVE9* overexpression) and treated them with IL-31 for 24 hours, we observed that the induction of profibrotic genes was attenuated (Figure 15, B and C). These results verified that SARA regulates and prevents pericyte-myofibroblast transdifferentiation mediated by IL-31 stimulation.

Interaction between cluster 12 noncanonical pericytes and lymphocytes was also suggested. Cells in cluster 12 strongly expressed the *Lgal1* gene, a well-known inducer of lymphocyte T apoptosis (68) (Supplemental Figure 6). In *SARAWT* mice treated with bleomycin, the *Lgal1*-expressing cluster was depleted, leading to the abnormal activation of T cells. Importantly, in bleomycin-treated *SARATg* mouse skin, cluster 12 and *Lgal1* expression were preserved, suggesting that this cluster prevents the Th2 cell expansion, the M2 macrophage polarization, and consequent fibrogenic changes.

### Effects of pericyte-specific, inducible SARA overexpression on skin fibrosis.

The data reported so far were generated using a *PDGFRb-Cre SARATg* mouse, where Cre recombinase was constitutively expressed under the control of *PDGFRb* promoter. As our data show, in agreement with literature (47), the *PDGFRb* promoter is active during embryogenesis in pericytes as well as inflammatory cells, revealing the interesting involvement of SARA in immune cell activation and in pericyte transdifferentiation. However, the Cre recombinase activity in both pericytes and inflammatory cells in the *PDGFRb-Cre SARATg* mice raised a possibility that antifibrotic effects we observed with the *PDGFRb-Cre SARATg* mice could be due to SARA overexpression in inflammatory cells in addition to pericytes. To test the specificity of the effect of SARA expression in pericytes, we repeated the experiments using an inducible Cre system (*PDGFRb-CreERT2*) (Supplemental Figure 7). In this mouse, Cre recombinase is expressed only after tamoxifen treatment after birth and therefore is active specifically in pericytes but not in inflammatory cells in which *PDGFRb* promoter is active only during embryogenesis (47).

**Figure 16:**
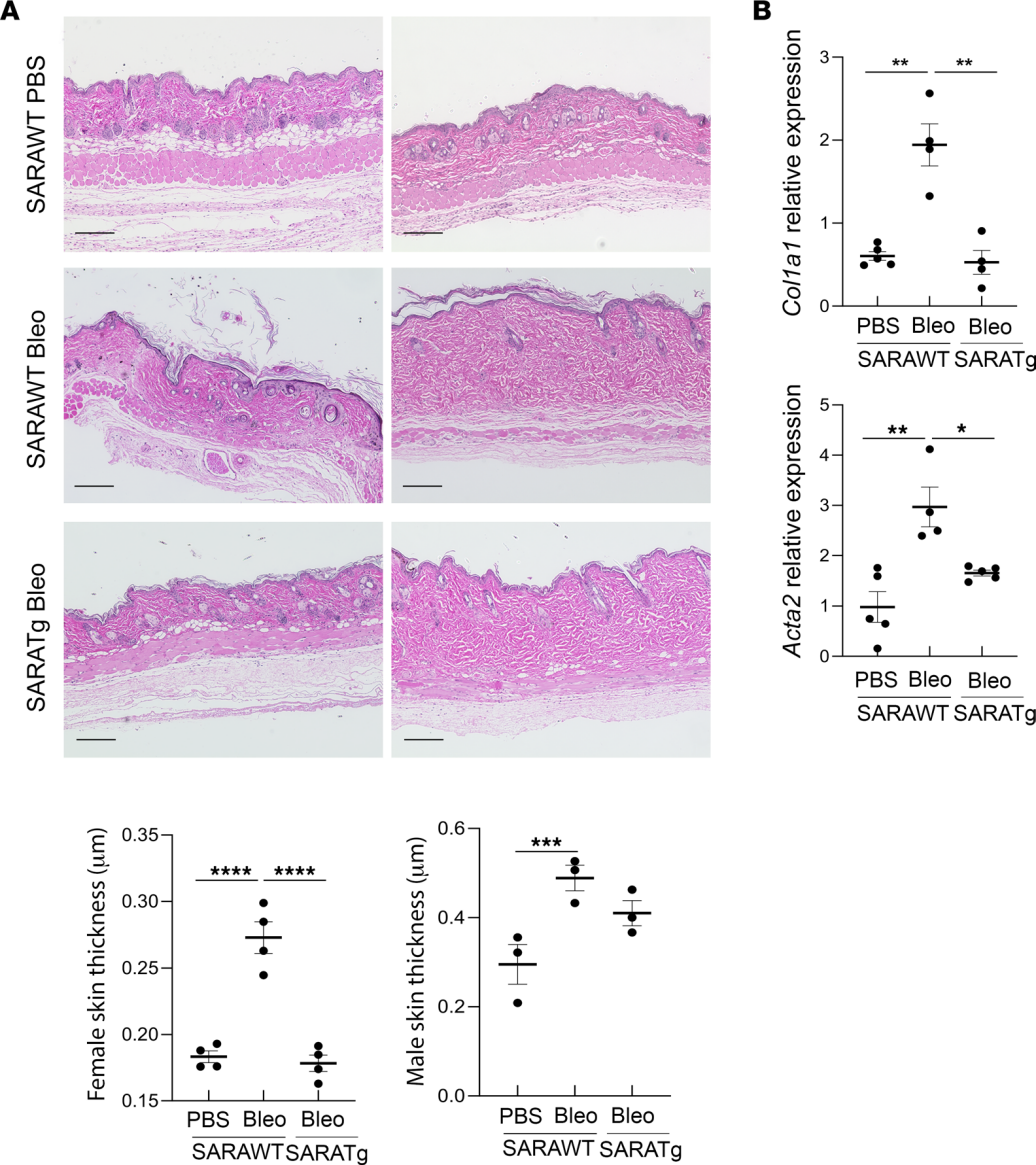
Effects of pericyte-specific inducible *SARA* overexpression on skin fibrosis. Representative images of H&E staining and dermal thickness measurement from female and male skin of tamoxifen-inducible Cre animal model, *PDGFRb-CreERT2/GFP/SARATg* or *WT*, subjected to PBS or bleomycin are shown. (**A**). Each dot represents the expression level from a different mouse. mRNA expression for profibrotic gene *Col1a1* and for activated myofibroblast marker *Acta2* in skin tissue evaluated by quantitative PCR is shown (**B**). Each dot represents the expression level from a different mouse. *SARAWT* animals *n* = 14 (PBS treated *n* = 7 and bleomycin treated *n* = 7) versus *SARATg* mice *n* = 7 (bleomycin treated *n* = 7). One-way ANOVA followed by Tukey’s multiple comparisons test: *P < 0.05, **P < 0.01, ***P < 0.001, and ****P < 0.0001.

Bleomycin-induced skin fibrosis was significantly less severe in the inducible *SARA*-overexpressing mice, suggesting that the protective effect of SARA was specific to pericytes, especially in females, and this was independent from the role of *SARA* overexpression in the inflammatory cells. In male mice, the protective effect of SARA on skin morphology was not as prominent as that observed in the constitutive Cre model, but the levels of *Col1a1* and *Acta2* mRNA in skin were still significantly reduced (Figure 16). These results suggest that *SARA* overexpression in the inflammatory cells may provide additional protection in fibrosis.

## Discussion

The data reported in this paper highlight potentially novel concepts in the pathogenesis of SSc. In this paradigm, SARA plays a key role. First, we show that some subsets of pericytes become myofibroblasts during fibrogenesis in a mouse model of SSc and that the overexpression of *SARA*, in PDGFR-β^+^ cells, protects against this transdifferentiation, hence preventing disease progression. Second, we show that signals associated with Th2 and macrophage activation are upregulated in the model and could be a therapeutic target. Our data also implicate a potentially novel crosstalk between immune cells and pericytes in the myofibroblast precursor transdifferentiation process.

Among several sources that have been implicated as myofibroblast precursors in skin fibrosis, pericytes are one of the most promising (10–13). Our findings are in agreement with the recent studies that have implicated pericytes as a source of myofibroblast progenitors in fibrosing organs (11, 69–73). Using genetic tracing approaches and kinetic analysis, Humphreys et al. first showed that most myofibroblasts derive from interstitial pericytes/perivascular fibroblasts in fibrotic kidney and proposed that vascular injury is the likely trigger for pericyte migration and differentiation into myofibroblasts (73, 74). In patients with dermal scarring, cells that express pericyte markers are found in the dermal layer, not alongside vascular structures, and they are positive for α-SMA and other ECM markers (75). Patients with SSc are also noted to show an increase in the number of cells positive for a pericyte marker, RG5, in the dermal layers of their skin samples, with a simultaneous upregulation in myofibroblast markers (76). These findings are further supported by data with inducible genetic fate mapping, which shows that a subset of stromal cells expressing ADAM12 are activated during injury in the dermis, and they represent the majority of collagen-producing cells during scarring. Interestingly, those cells are also PDGFR-β^+^ and NG2^+^, developmentally different from other skin cells, and derived from fetal cells involved in vascular development (70). PDGFR-β^+^ cells are also identified as the main population that increases during spinal cord scar formation, with a consistent upregulation of α-SMA and fibronectin expression (77). Hepatic stellate cells, liver-specific pericytes, are also the principal collagen-producing cells in liver fibrogenesis (78). These findings suggest that pericytes serve as precursors for myofibroblasts in many organs other than skin as well and therefore a promising target in treating organ fibrosis.

Our data suggest a potentially novel function of SARA in 2 ways, one in pericytes and one in inflammatory cells, with the final effect to regulate pericyte cell identity. SARA is a highly conserved protein, originally reported as a modulator of the TGF-β pathway. However, subsequent works by us and others showed that SARA is indeed dispensable for TGF-β signaling (30, 31, 33) and defined a role of SARA independent of the TGF-β pathway, including in intracellular molecular trafficking (27) and neuronal development (29, 32). We have previously demonstrated that SARA is critical in maintaining epithelial cell phenotype in culture. SARA levels are higher in cultured kidney epithelial cells than in fibroblasts and inversely correlated with α-SMA expression level. Treatment with TGF-β1, a well-known mediator of fibrosis, decreased SARA levels at the same time frame of mesenchymal marker upregulation. Moreover, silencing SARA expression resulted in α-SMA upregulation and mesenchymal phenotype, even without TGF-β1 stimulation (26). Consistent with these in vitro findings, we show in the present study that mice overexpressing *SARA* in PDGFR-β cells showed significantly less skin fibrosis upon bleomycin treatment compared with *SARAWT* littermates. *SARA* overexpression in PDGFR-β cells with a constitutively active Cre was effective in preventing fibrosis in both female and male mice, even though males have a physiologically thicker dermis and bleomycin induced severer changes. However, the protective effect was less apparent in males when an inducible Cre, which limits *SARA* overexpression exclusively in pericytes but not in inflammatory cells, was used. SSc primarily affects females, but males present with more severe fibrotic changes when affected, and males have higher mortality than females (79).

Our results suggest that loss of SARA expression in inflammatory cells might be an aggravating factor for SSc. Indeed, it has been described that the expression of SARA in Th1 and Th2 cells is decreased during the activation of the lymphocytes, and consequently the responsiveness to TGF-β is increased (80, 81).

scRNA-Seq technology unveiled heterogeneity of the cells in healthy and pathological conditions. A scRNA-Seq analysis of cells in human fibrotic skin showed that fibroblasts are heterogeneous (82) and that the fibroblasts of mesenchymal origin were significantly increased in keloid compared to normal scar, as well as in SSc (82). Some of the fibroblast clusters found in SSc skin were most analogous to mesenchymal fibroblasts in keloid, implicating a common mechanism in fibrotic skin disease (82). It is well known that endothelial cells demonstrate pathological changes in the early stage of the disease and drive the other clinical manifestations of SSc. Indeed, another scRNA-Seq analysis revealed that genes associated with vascular injury, ECM assembly/organization, and negative regulation of angiogenesis are upregulated in endothelial cells from patients with SSc (83). Since pericytes represent only a small fraction of cells in skin, previous studies using cells isolated from the entire skin or dermal layer did not have enough power to characterize subpopulations of pericytes. In the present study, we isolated PDGFR-β^+^ cells to focus our scRNA-Seq analysis on pericytes as a potential source of myofibroblasts and identified 6 distinct clusters within the pericyte population. In addition to pericytes that express the canonical markers, we identified a subset of pericytes that do not express canonical pericyte markers but are characterized by expression of *Mfap5* and *Cthrc1* as specific pericyte subpopulations that assume myofibroblast characteristics. CTHRC1 is a secreted protein that has hormone-like characteristics and is known to regulate tissue remodeling processes and blood vessel formation. MFAP5 is involved in maintaining vessel integrity (48, 49) and also reported to promote epithelial-mesenchymal transdifferentiation (EMT) program in basal-like breast cancer (84). Activation of EMT is triggered by a series of paracrine factors such as NOTCH, WNT, and TGF-β, and they all synergize each other to induce the phenotype (85). STRING analysis shows, in fact, that MFAP5 and CTHRC1 have a functional relationship with NOTCH receptors and ligands and WNT signaling, respectively. Indeed, MFAP5 and CTHRC1 modulate EMT via NOTCH and WNT, respectively (86–88), and SARA might contribute to modulate these pathways.

Immunostaining experiments on mouse skin tissue supported the involvement of these pericyte subsets, and SARA overexpression protected them to transdifferentiate toward myofibroblasts. In addition, SSc patient skin samples clearly showed an activation of those cells, showing the clinical relevance of this finding.

In our scRNA-Seq data set, we found that SARA overexpression, in PDGFR-β^+^ cells, changes the trajectory of pericytes’ path in fibrotic conditions, modulating the state of the cells and their transdifferentiation, further suggesting that SARA negatively regulates the phenotypic differentiation via suppressing the signal of the profibrotic molecules *Nme2* and *Gas5* and upregulating the antifibrotic mediator *Cyr61*. We highlight that in fibrotic conditions those pericytes are probably partially and not completely transdifferentiated; indeed, the transdifferentiation is not an on-off process but a dynamic evolution where the entire spectrum of transdifferentiation degrees can exist, and SARA overexpression modulates this dynamic, not completely reverting the change observed in the *SARAWT* mice.

In vitro experiments with human pericytes supported the modulation of these molecules’ expression level after SARA downregulation or overexpression under fibrotic stimuli, such as TGF-β treatment.

In SSc skin, an unbalanced activation of the immune system is characteristic (89–91), and diverse immune cell types are implicated in the immunopathogenesis of SSc, including T cells, B cells, dendritic cells, mast cells, and macrophages. SSc patients show a higher percentage of activated T cells (91, 92), and Th1/Th17-driven immune response is the first phase of SSc, followed by a switch to Th2-driven immune response, leading to irreversible fibrosis. In the bleomycin model, recruitment of mast cells increases by week 1 followed by production of cytokines by week 2, and dermal thickness gradually increases to maximize by week (42, 43, 93). In the pathogenesis of SSc, TGF-β plays one of the main roles and TGF-β levels are elevated (94). TGF-β not only modulates EMT and myofibroblast activation but also affects the proliferation of Th1 lymphocytes, but not of Th2 lymphocytes (95), resulting in preferential activation of Th2 cells. In our *PDGFRb-Cre* model, Cre activity was detected in pan-leukocytes in addition to pericytes presumably due to the activation of this promoter in leukocyte progenitors during embryogenesis (47). In particular, a cluster enriched with cells from *SARAWT* mice treated with bleomycin showed significant upregulation of *Retnla* expression. RETNLA is a molecular product of the Th2 signaling pathways, and IL-31 is one of the most highly produced cytokines of activated Th2 lymphocytes (96). A profibrotic function of IL-31 has also been reported, and it may contribute to skin and lung fibrosis in a subset of patients with SSC (97). A recent study that demonstrated IL-31–mediated Th2 polarization leads to fibrosis provides another rationale for targeting the IL-31/IL-31RA axis in the treatment of SSc (98). IL-13 and IL-4 are also primarily produced by Th2 cells. IL-13 and its receptor expression levels are elevated in a murine model of bleomycin-induced scleroderma (99). IL-13 levels are also elevated both in the blood and in the skin of patients with SSc (92). IL-4 and IL-13 are also considered possible therapeutic targets in SSc (100, 101). We harvested cells 3 weeks after the start of the bleomycin injection; therefore, molecular profiles we obtained may lack some of the early inflammatory signals but are more relevant to a clinical setting where patients are often left undiagnosed till overt skin fibrosis develops (1). Mouse pericytes treated with IL-31 clearly showed profibrogenic activities, which was more emphasized after SARA downregulation and attenuated after SARA overexpression, highlighting how SARA modulated the pericyte capability to transdifferentiate, acting with different mechanisms. Our results suggest that SARA exerted its antifibrotic effects by at least 2 distinct mechanisms, one via prevention of Th2 cell activation and macrophage polarization and the second via a direct effect on pericytes independent of inflammatory cells. Pericyte-specific *SARA* overexpression with an inducible Cre showed complete restoration of normal skin morphology in female mice but not in males, supporting that this dual mechanism of action could at least partially explain the difference in SSc progression seen between males and females in the clinical setting.

Our scRNA-Seq data also suggested that specific immune signals regulate myofibroblast precursor transdifferentiation, and conversely myofibroblast precursors modulate immune system activation. The 2 clusters identified in our work, cluster 10 and 12, seem to modulate each other, underlining regulation of the cell phenotypes and functions by microenvironment cell-cell communication in developing more effective therapeutic strategies.

In addition to pericytes and immune cells that consisted of 2 major populations of PDGFR-β^+^ cells we isolated and analyzed by scRNA-Seq, we noted a few clusters of endothelial cells and keratinocyte populations. Since they did not change regardless of presence of fibrosis and/or SARA overexpression, it is unlikely that these clusters contributed to the phenotype of the PDGFR*b*-Cre *SARATg* mice. Some fibroblasts also expressed PDGFR-β. However, we did not find a cluster that was signified as fibroblasts in our scRNA-Seq data set. Some of the noncanonical pericytes we identified could be innate fibroblasts. We previously showed that low SARA expression is one of the characteristics for fibroblasts at least in culture (26), and therefore SARA overexpression that might have happened in at least a portion of fibroblasts in this model could have contributed to the phenotype of our model.

The data presented in this paper show a potentially novel antifibrotic role for SARA via preventing the pericyte-myofibroblast transdifferentiation and aberrant lymphocyte activation in SSc. They also suggest a potentially novel crosstalk between Th2 cells, macrophages, and pericytes, in which SARA plays a critical role. The results shown in this report are promising for the development of treatment options for SSc.

## Methods

Detailed materials and methods are provided in Supplemental Methods.

### Mice.

*PDGFRb-Cre* (40) and *Z/EG* (46) mice were gifts from RF Adams (London Research Institute, London, United Kingdom) and CG Lobe (Sunnybrook and Women’s Health Science Center, Toronto, Ontario, Canada), respectively. *PDGFRb-CreERT2* (stock 030201) and *C57Bl/6J* mice (stock 000664) were purchased from Jackson Laboratories.

### Generation of SARATg mice.

Full-length 4.6 kb *ZFYVE9* cDNA that encodes human SARA protein was subcloned from TOPO vector and inserted into pWT326 that contains *CAG* promoter preceded by a lox-stop-lox cassette (102) at ClaI/SalI, then subcloned to a *Rosa26-1* targeting vector, *pWT242* (103) at PacI/AscI, and injected into RI-129 ES cells (104). Positive clones were selected by Southern blot screening, then surgically transferred into the oviduct of pseudo-pregnant females. *SARATg* mice were mated either with *PDGFRb-Cre* or *PDGFRb-Cre-ERT2* mice, genotyped using ReadyMix Taq reaction mix (MilliporeSigma), and then further mated with *Z/EG* mice to express GFP in PDGFR-β^+^ cells. The mice were subjected to experiments at age 8 weeks. Primers used for genotyping are shown in Table 4.

### Induction of CreERT2 recombinase activity.

*PDGFRb-CreERT2 Z/EG SARATg* mice were given tamoxifen (T5648, MilliporeSigma) (2 mg daily) intraperitoneally, 5 times a week for 2 consecutive weeks at the age of 6 weeks old, to induce Cre recombinase activity. Recombination and expression of the transgene were validated and are shown in Supplemental Figure 7.

### Induction of skin fibrosis.

Skin fibrosis was induced by daily subcutaneous injection of bleomycin (20 mg/kg, body weight) at shaved back skin for 2 weeks. Mice were sacrificed 1 week after the completion of bleomycin injection, and skin samples were harvested for histological evaluation and molecular analyses.

### scRNA-Seq.

PDGFR-β^+^GFP^+^ cells from back skin harvested from *PDGFRb-Cre Z/EG*, *SARATg*, or *SARAWT* mice, subjected to bleomycin or PBS, were sorted by flow cytometry and subjected to scRNA-Seq. Sequencing of the 10x Genomics single-cell libraries was performed on the Core’s Illumina HiSeq 4000, at the depth of ~30,000 reads per cell. The analyses were performed by the bioinformatician of Sequencing Core Facility of the Robert H. Lurie Comprehensive Cancer Center of Northwestern University. The details are provided in Supplemental Methods.

### Data sharing.

scRNA-Seq raw data used in the above analyses are uploaded to NCBI GEO database under accession number GSE211810.

### Statistics.

Statistical analyses were performed using GraphPad Prism version 8 software (GraphPad Software) for 2-tailed Mann-Whitney test or Kruskal-Wallis 1-way ANOVA followed by post hoc analysis. The data are reported as mean ± SEM. Statistical significance was defined as *P* < 0.05.

### Study approval.

All experiments involving animal use were performed in accordance with the *Guide for the Care and Use of Laboratory Animals* of the NIH (National Academies Press, 2011). The protocol was approved by the Institutional Animal Care and Use Committee (protocol IS00005976).

Deidentified skin tissue was obtained from the Northwestern University Skin Tissue Engineering and Morphology Core, collected under an approved protocol in compliance with the Northwestern University Internal Review Board (IRB STU00009443). This work did not constitute human subject research.

## Author contributions

KCS, JV, and TH performed conceptualization. KCS, ICLP, and TH developed methodology. KCS, XL, and VD performed investigation. KCS, XL, and TH performed visualization. TH acquired funding. ICLP, JV, and TH performed supervision. KCS wrote the original draft. JV, ICLP, and TH reviewed and edited the draft.

## Supplementary Material

Supplemental data

## Figures and Tables

**Table 4:**
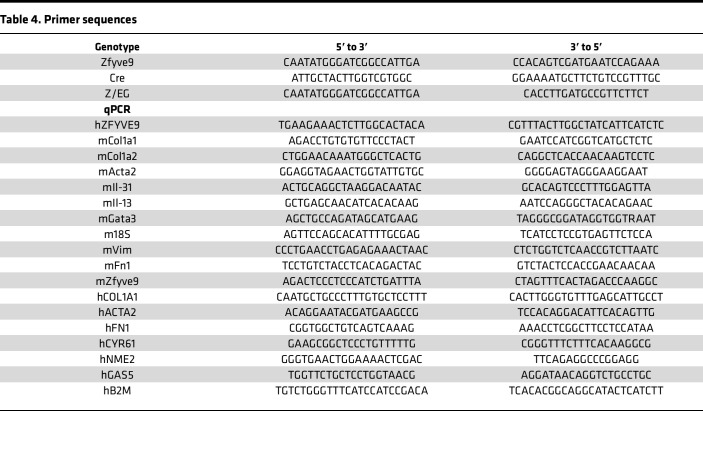
Primer sequences

**Table 3:**
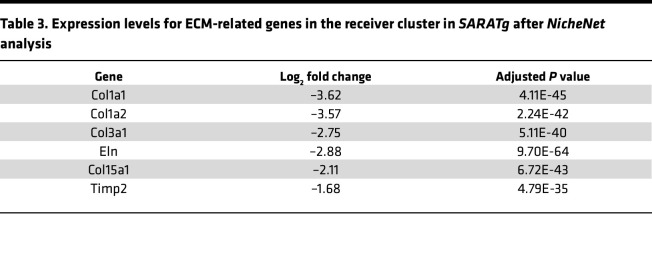
Expression levels for ECM-related genes in the receiver cluster in *SARATg* after *NicheNet* analysis

**Table 2:**
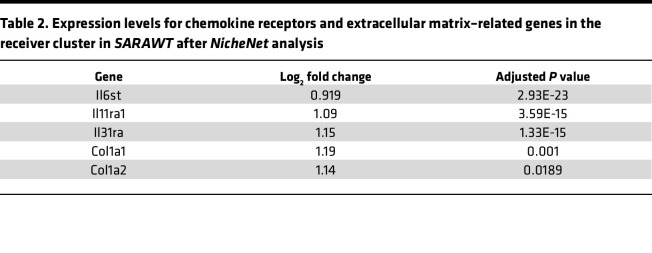
Expression levels for chemokine receptors and extracellular matrix–related genes in the receiver cluster in *SARAWT* after *NicheNet* analysis

**Table 1:**
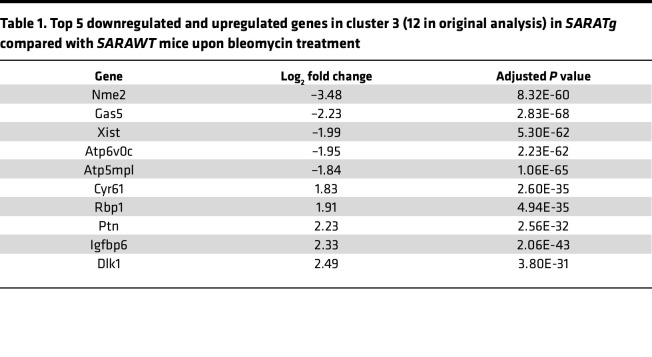
Top 5 downregulated and upregulated genes in cluster 3 (12 in original analysis) in *SARATg* compared with *SARAWT* mice upon bleomycin treatment

## Version 1 09/22/2022

In-Press Preview

## Version 2 11/08/2022

Electronic publication
