# Supplementary material for: SARA suppresses myofibroblast precursor transdifferentiation in fibrogenesis in a mouse model of scleroderma
Source: JCI Insight. 2022 Nov 8;7(21):e160977. doi: 10.1172/jci.insight.160977 (PMC9675568; doi:10.1172/jci.insight.160977)
Supplement: Supplemental data [file jciinsight-7-160977-s151.pdf]

SUPPLEMENTARY MATERIAL

DETAILED MATERIALS AND METHODS

Histological evaluation and skin thickness measurement

Tissues were fixed in 10% neutral buffered formalin for at least 48 hours, embedded in paraffin and sectioned at 4 μm thickness. Hematoxylin eosin (H&E) staining was performed with standard protocols by the Mouse Histology and Phenotyping Laboratory of the Northwestern Core Facility. Images of the sections were then acquired with the Keyence BZ- X800 series microscope (Keyence, Osaka, Japan). either with 10X and 20X objectives and the degree of fibrosis was evaluated by dermal thickness measured with ImageJ software. Six random areas of each skin section, captured with a 20X objective lens, were measured and the average of the measurements was used as a data point for each animal. The analysis was conducted separately for female and male samples, because of the physiological sex-dependent differences in skin morphology.

Immunofluorescence and confocal microscopy

Skin sections were deparaffinated and rehydrated, antigens were retrieved by steaming with Target Retrieval Solution (DAKO/Agilent Technologies, Carpinteria, CA). Nonspecific bindings were blocked with 1XPBS/1%BSA/0.1% TritonX-100 for 1h at RT. Sections were incubated with primary antibodies overnight at 4ºC and followed by incubation with secondary antibodies, when needed, for 1h and 30minutes at 4ºC. DAPI was used for nuclei staining. Antibodies used were; anti-NG2 (MA5-24247, ThermoFisher, Waltham, MA) (1:75), anti-αSMA (ab5694, abcam, Cambridge, UK) (1:150), anti-CD31-Alexa647 (102416, BioLegend, San Diego, CA) (1:75), anti-CD45 (ab23910, abcam, Cambridge, UK) (1:100), anti-CD146-APC (50-1469-42, ThermoFisher, Waltham, MA) (1:40), anti-IL-31 (MA5-34912, ThermoFisher, Waltham, MA), anti-pSTAT3 (ab267373, abcam, Cambridge,

UK), anti-SARA (14821-1- AP, Proteintech, Rosemont, IL), anti-MFAP5 (15727-1-AP, Proteintech, Rosemont, IL) and anti-CTHRC1 (16534-1-AP, Proteintech, Rosemont, IL) . The secondary antibodies used were the following: goat anti- Rat-APC (SAB4600186, Sigma-Aldrich, St. Louis, MI) (1:200), goat-anti-Mouse-Alexa568 (A11031, Molecular Probes, Eugene, OR), and goat anti-Rabbit-Alexa568 (A11011, Thermo- Fisher, Waltham, MA) (1:200). Images of the sections were acquired with Nikon A1 confocal laser microscope system (Nikon, Tokyo, Japan) at the Center for Advanced Microscopy/Nikon Imaging Center of Northwestern University. Negative control images with rabbit or mouse IgG and secondary antibody are shown in S2B.

The quantification of NG2+/αSMA+ cells was performed using TissueQuest software (TissueGnostic, Vienna, Austria).

The corrected total cell fluorescence (CTCF) for NG2, αSMA, CD45, IL-31, pSTAT3 and SARA was quantified using image J software.

Collagen quantification

Five to ten 10 μm skin tissue sections were transferred into Sarstedt tubes and hydrolyzed by addition of 150 μl 6M HCl incubation at 95°C over-night in a heat block. After hydrolysis, 35 μl were used for collagen quantification using the QuickZyme total collagen (QZBTOTCOL1, QuickZyme Biosciences, Leiden, The Netherlands) assay following manufacture instructions. Protein concentration of the lysate was measured with 15 μl of the lysate using the QuickZyme protein assay (QZBTOTPROT1, QuickZyme Biosciences, Leiden, The Netherlands), which is specifically designed for protein analysis in acid hydrolysates. The colorimetric product of the sample was read at 570 nm using a plate reader, and the reading was corrected by the protein concentration.

In addition, Masson's thricrome staining was performed on skin sections by Northwestern University Mouse Histology & Phenotyping Laboratory (MHPL).

RNA isolation and qPCR

Total RNA was extracted from skin tissue using the RNeasy mini kit (74104, Qiagen, Valencia, CA) following the manufacturer instructions. 1 μg of RNA, quantified with a Nanodrop, was reverse transcribed with the iScript cDNA synthesis kit (1708891, Bio-Rad Laboratories, Hercules, CA) and subjected to quantitative PCR using the iTaq Universal SYBR Green Supermix (1725121, Bio-Rad Laboratories) with the CFX96 Real-Time System (Bio-Rad Laboratories). Real-time data were collected for 45 cycles of 95°C, 5 s; 60°C, 30 s. Primers used are custom synthesized by Integrated DNA Technology (Coralville, CA) and their sequence reported in Table 1. Relative expression of the genes of interest was evaluated using the ΔΔCt method (1) with 18S and β2m as a reference gene. Samples were analyzed in triplicate, and experiments were repeated at least three times.

Cell isolation from tissue and flow cytometry

Back skin harvested from *PDGFRβ-Cre;Z/EG; SARA*$^{Tg\ or\ WT}$ mice, subjected to bleomycin or PBS, was washed in cold PBS twice and minced using razor blades and dissecting scissors in a sterile dish on ice until the sample was of a uniform consistency with 2-3 mm pieces, then digested with Liberase TM (Millipore Sigma, 0.5 mg/ml in DMEM/F12) containing DNase (Millipore Sigma, 100U/ml) for 1h and 30minutes at 37°C in rotation. The reaction was stopped by addition of fetal bovine serum (FBS) (10% by volume of the reaction mixture). Undigested tissue pieces were filtered by 100μm on-tube filter, and cells were pelleted and resuspended in flow cytometry buffer (PBS ,1%FBS, 0.5% BSA). If there was a significant red blood cell contamination, the pellet was resuspended in RBC lysis buffer (00-4300-54, Thermo Fisher, Waltham, MA) 1X and incubated for

5 min at room temperature. After washing the pellet with 1XPBS, the samples were incubated for 30 minutes with antibodies at 4ºC, followed by 20 minutes incubation with the secondary antibody when needed. Cells were then washed and resuspended with flow cytometry buffer. The cells were stained with anti-NG2 (MA5-24247, Thermo Fisher, Waltham, MA) (1:75) and anti-CD45-APC-Cy7 (47-0451-82, Thermo Fisher, Waltham, MA). The secondary antibody used for NG2 detection was goat anti-Rat-APC (SAB4600186, Sigma-Aldrich, St. Louis, MI) (1:200). One µl of DAPI (D3571, Thermo Fisher, Waltham, MA) was added to the cell suspension to detect dead cells immediately prior to flow cytometry that was performed on a BD FACS Aria SORP system (BD Biosciences, San Jose, CA).

Single Cell RNASeq Library Preparation and Sequencing

Samples for single-cell RNASeq were examined first for cell viability, density, and prep quality ensuring no clumps, cell debris, etc. Samples passing the QC process were loaded onto 10x**.** Genomics Chromium for partitioning and encapsulation of single cells into nanoliter-sized GEMs (Gel beads-in-EMulsion). Each encapsulated cell was then lysed within its GEM. The total RNA released was reverse transcribed to cDNA with primers attached to the gel bead, each of which carries a unique 10x barcode for downstream cell separation. Subsequently the GEMs were broken, and all uniquely barcoded cDNAs were pooled and then PCR amplified to generate enough material for Illumina sequencing. This sequencing library construction process was carried out using the 10X Genomics Single Cell 3' v3.1 Protocol. Sequencing of the 10x libraries was performed on the Core's Illumina HiSeq 4000, at the depth of ~30,000 reads per cell.

Single Cell RNASeq Analysis

Raw sequencing data, in base call format (.bcl) was demultiplexed using Cell Ranger (version 4.0.0) from 10x Genomics, converting the raw data into FASTQ format. Cell Ranger was also used for aligning the FASTQ files to the mm10 reference genome provided by 10X Genomics and also for counting the number of reads from each cell that align to each gene. The matrix files and feature files summarizing the alignment results were analyzed in Seurat (Satija Lab, NYGC) for further analysis. In Seurat, each individual sample was preprocessed, normalized, and scaled. Each sample underwent quality control measures to check for the number of genes, UMIs, and percent mitochondrial genes detected per cell, and appropriate filters were used to remove any outlier cells. All samples were combined into a single dataset, adding metadata with the original sample information. This combined dataset was used for downstream analyses including finding biomarkers and differential expression comparisons between specific clusters.

The pathway enrichment analysis was performed using the g:GOSt functional profiling tool available on the g:Profiler web server (version e99_eg46_p14_f929183) to identify significant pathways among the clusters. Gene Ontology (GO), Kyoto Encyclopedia of Genes and Genomes (KEGG) and Reactome (REAC) databases were used as sources.

Cell communication was explored using *NicheNet* (2, 3). The combined Seurat object containing all groups was used as the underlying dataset with Cluster 10 as the sender cell population and Cluster 12 as the receiver cell population. Communication was studied between groups SARA^{WT} treated with PBS or bleomycin and between groups SARA^{Tg} treated with PBS or Bleomycin.

To construct single-cell pseudotime differentiation trajectory, the Seurat single-cell dataset was converted into a Monocle dataset using the Monocle3 conversion tool "as.cell_data_set()".

A trajectory analysis was then performed using the standard Monocle3 workflow (http://cole-trapnell-lab.github.io/monocle-release/docs/#constructing-single-cell-trajectories) (4-7).

Enzyme-linked immunosorbent assay (ELISA)

Soluble protein lysate was prepared with mouse skin tissue for IL-31 detection and quantification using the Mouse IL-31 ELISA Kit (Abcam, Cambridge, UK). Briefly, samples were loaded onto a 96-well microplate coated with the capture anti-mouse IL-31 antibody for 90 minutes at 37°C. The plate content was discarded, and the biotinylated detection antibody was added for 60 minutes at 37°C. After three washes, the Avidin-Biotin-Peroxidase Complex (ABC) solution was added to the samples for 30 minutes at 37°C. Upon other three washes, the samples where incubated for 30 minutes in the dark with 3,3′,5,5′-Tetramethylbenzidine (TMB) substrate solution. The sample OD was read at 450 nm using a plate reader. Total protein concentration, using a Bicinchoninic acid assay (BCA), was used to normalize the IL-31 amount detected.

Mouse pericyte culture and IL-31 treatment

Gli1+ pericyte that were isolated from Gli1-Cre; tdTomato; Immortomouse (8) was obtained from Dr. Humphreys (University of St. Louis, St. Louis, MO) and the cells were cultured at 37ºC, 5% CO2 with α-MEM glutaMAX media (ThermoFisher, Waltham, MA) containing 20% MSC-FBS (ThermoFisher, Waltham, MA), 10ng/ml mouse basic fibroblast growth factor (Gemini Bio-Products, West Sacramento, CA), 10ng/mL mouse epithelial growth factor (Gemini Bio-Products, West Sacramento, CA) and 1% Penicillin/Streptomycin. For in vitro studies they were treated with 50ng/ml mouse IL-31 (210-31, PeproTech, Cranbury, NJ) for 8h, 24h and 48h. After the time course, the cells were fixed in 3.7 % formaldehyde and processed for immunofluorescence or submitted to RNA extraction for molecular analyses.

Human pericyte culture and TGFβ treatment

Human placental pericytes were purchased from PromoCell (C-12980, PromoCell GmbH, Heidelberg, Germany) and they were cultured in Pericyte Growth Medium 2 supplemented with SupplementMix (C-28041, PromoCell GmbH, Heidelberg, Germany) at 37ºC, 5% CO2. For in vitro study they were treated with 2ng/ml Recombinant Human TGFβ1 (240–B, R&D system, Minneapolis, MN) for 24h and RNA was extracted for molecular analyses.

siRNA transfection

Mouse and human pericytes were transfected with 50 nM of siRNA against *Zfyve9* (J-050541-09-0002, Horizon, Cambridge, UK) or *ZFYVE9* (J-011939-05-0002, Horizon, Cambridge, UK) respectively, or 50 nM of a non-targeting siRNA (D-001810-01-05, Horizon, Cambridge, UK) in antibiotic-free complete medium. Dharmafect 2 (T-2002-03,Dharmacon, Horizon, Cambridge, UK) was used for transfection at 0.5 μL/100 μL medium following the manufacturer's instructions. 24 hours after transfection, medium was changed to serum-free, antibiotic-containing medium, and cells were treated with either IL-31 or TGFβ (and vehicle as negative controls) for 24 hours as described above. Cells were then lysed for RNA extraction and molecular analyses. Target mRNA sequences for siRNAs used: *Zfyve9* –GUAUAAAGCAAACGGAAAA, *ZFYVE9*–GCUGUAGCCUGAAAUGUAA, non-targeting – UGGUUUACAUGUCGACUAA.

ZFYVE9 overexpression

SARA DNA (A gift from Dr. Wrana, Mt Sinai Hospital, Toronto, Canada), or an empty vector (PCDNA5) was transfected to the mouse and human pericytes using Lipofectamine (2 ml/1 mg of DNA) following the manufacturer's instructions (15338-100, ThermoFisher, Waltham, MA). One

day after the transfection, medium was changed to serum-free, antibiotic-containing medium, and cells were treated with either IL-31 or TGFβ (and vehicle for negative controls) for 24 hours as described above. Cells were then lysed for RNA extraction and molecular analyses.

SUPPLEMENTARY FIGURES

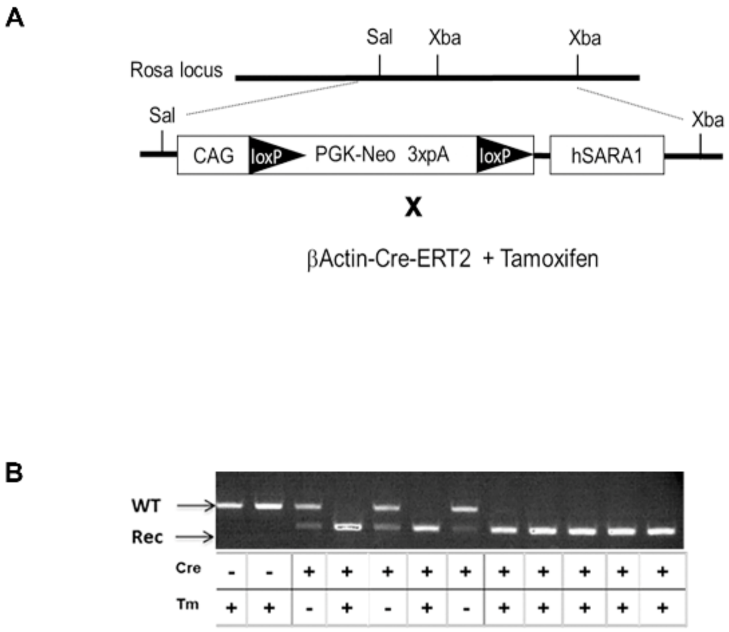

Fig. S1. Generation of *SARA*^{Tg} mouse. Construction of the targeting vector (A). Recombination was confirmed by crossing the *SARA*^{Tg} mice with the systemic and inducible Cre line (*βActin-Cre*^{ERT2}). Genotyping results of the *SARA*^{Tg} ; *βActin-Cre*^{ERT2}mice with or without tamoxifen induction are shown (B).

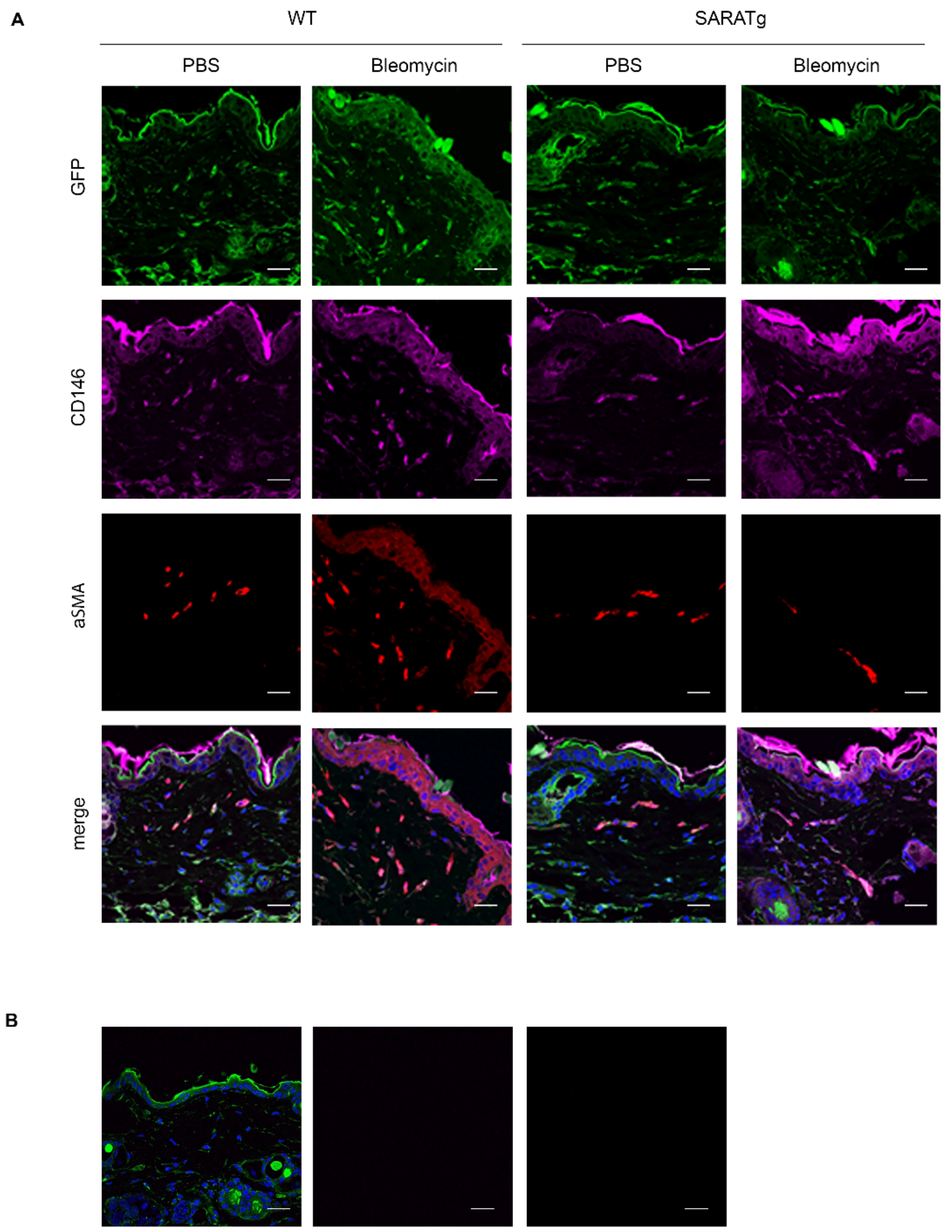

Fig. S2. Immunofluorescence for CD146 pericyte marker and αSMA in *SARA^{WT}* and *SARA^{Tg}* mice upon bleomycin injection.

Representative images of immunofluorescence staining on skin sections for another pericyte marker CD146 and myofibroblast marker αSMA are shown in A. As observed for NG2 as

well, CD146 was detected among GFP+ cells, showing the same pattern and changes observed for NG2 marker. Single channels and merge snapshots are shown in the panel. Scale bar = 20μm. Representative pictures from 3 independent experiments are shown. Negative control staining for NG2 and αSMA are shown in B. Immunofluorescence for CD146 and CD31 were performed using fluorophore-conjugated antibodies.

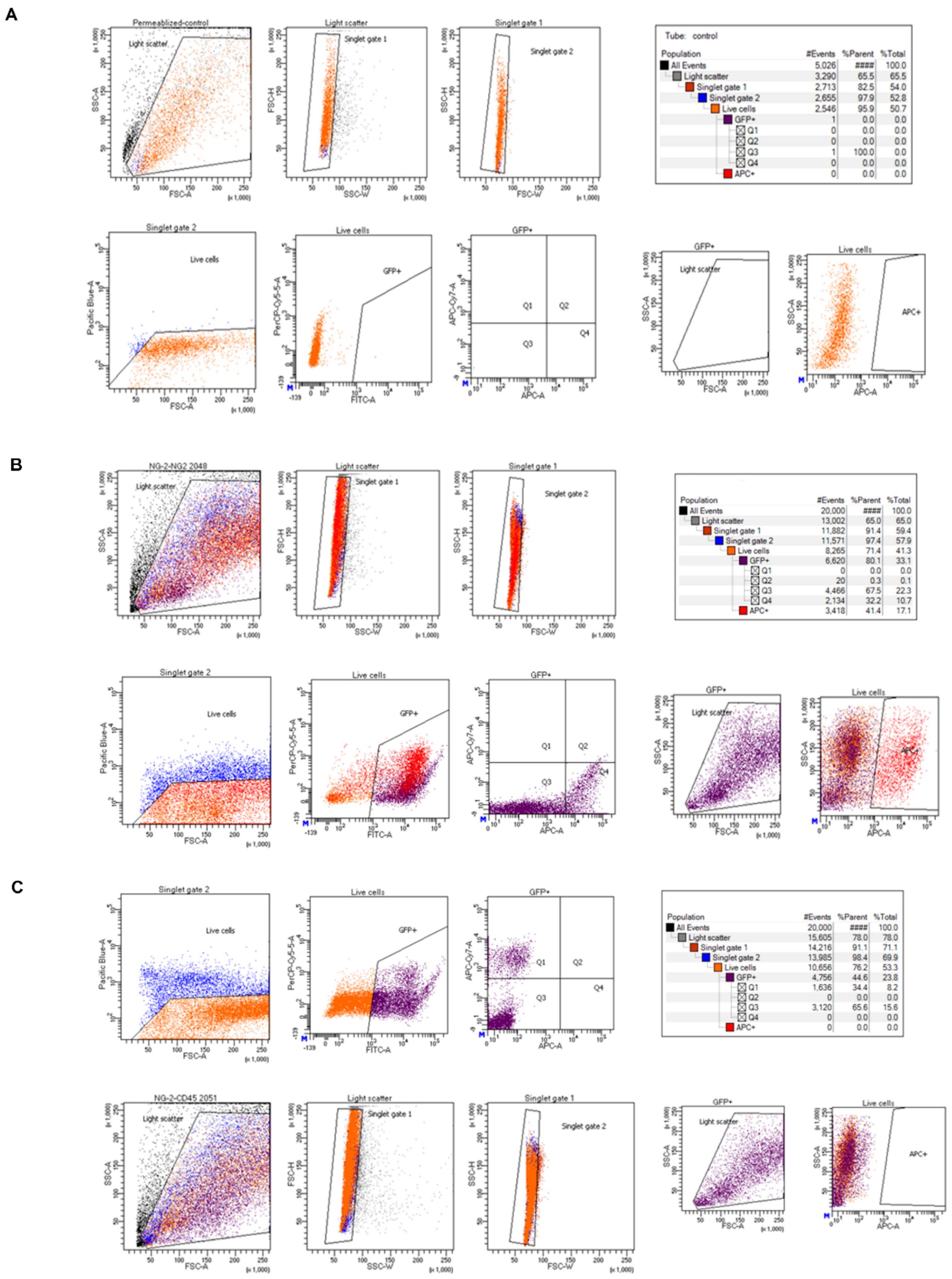

Fig. S3. Flow cytometry analysis on GFP+ cells in skin of the *PDGFRβ-Cre;Z/EG; SARA*$^{Tg\ or\ WT}$ mice. Cells isolated from *PDGFRβ-Cre;Z/EG;/ SARA*$^{Tg\ or\ WT}$ skin tissue were analyzed by flow cytometry to detect their identity, staining them for pericyte marker NG2 and pan-leukocyte

marker CD45. Control gating scatterplots are shown in A. Representative scatterplot of live GFP+/NG2+ cells (APC-conjugated antibody) (B) and representative scatterplot of live GFP+/CD45+ cells (APC/Cy7-conjugated antibody) (C) are shown.

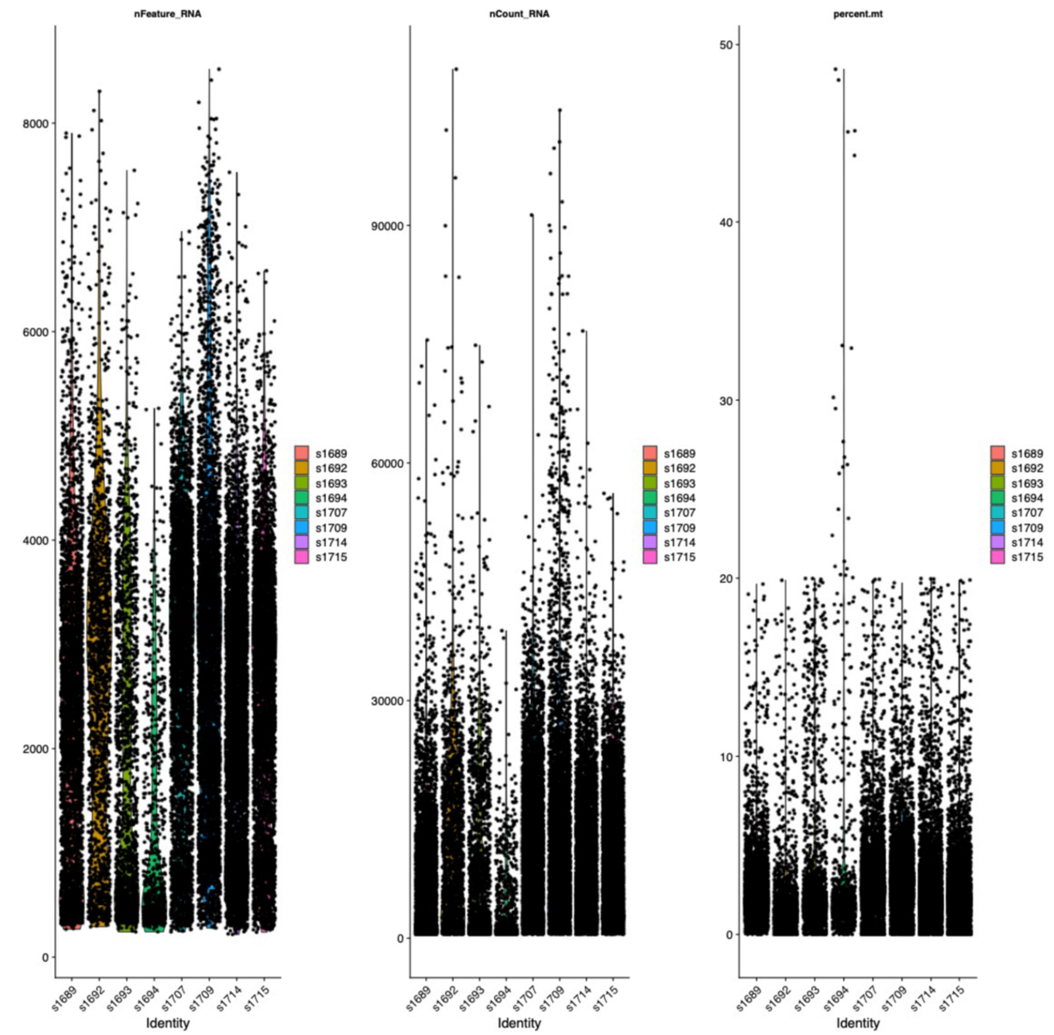

Fig. S4. Quality controls of scRNASeq analyses

Basic quality check for all the samples submitted to scRNASeq are shown. The violin plots show the nFeature_RNA, nCount_RNA and percent.mt of all the samples.

A

Normal skin ID	Year of Birth	Gender	Ethnicity	Year of sample collection
NUTC3866.19	1969	F	Caucasian	2019
NUTC3878.19	1976	F	Caucasian	2019
NUTC 4286.21	1983	F	Caucasian	2021
NUTC 3827.19	1949	F	Caucasian	2019
NUTC 3837.19	1961	F	Caucasian	2019
NUTC 3711.19	1979	F	Caucasian	2019
NUTC 3769.19	1987	F	Caucasian	2019
NUTC 3699.19	1971	F	African American	2019
NUTC 3838.19	1981	F	Caucasian	2019

DP Number	Year of Birth	Gender	Ethnicity	Year of sample collection
DP 2007-0006045	1962	M	White not Hispanic	2007
DP 2007-00010250	1975	F	"Other"	2007
DP 2007-0013010	1967	M	White not Hispanic	2007
DP 2007-0019666	1951	M	White not Hispanic	2007
DP 2007-0019927	1958	F	White not Hispanic	2007
DP 2008-0006389	1933	F	White not Hispanic	2008
DP 2008-0007376	1936	M	White not Hispanic	2008
DP 2008-0016207	1949	M	White not Hispanic	2008
DP 2009-0003890	1956	M	White not Hispanic	2009

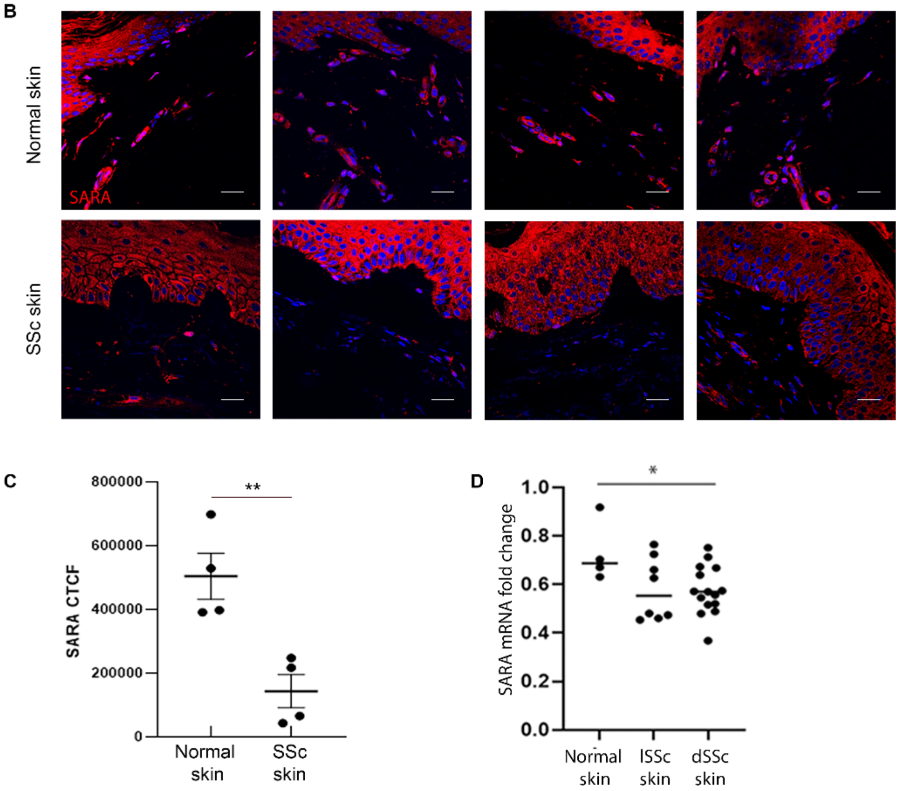

Fig. S5. Detailed information of the human skin biopsies and SARA in SSc patients

Demographic information of volunteer and disease patients (DP) are shown in the tables in A. Normal skin ID or DP number, year of birth, gender, ethnicity, and year of sample collection are reported.

Immunofluorescence staining for SARA in SSc skin biopsies. Representative images of SARA levels

in four different SSc skin biopsies, compared to normal skin, are shown in B. The corrected total cell fluorescence (CTCF) quantification is shown in the graph (C). Nuclei were detected with DAPI and the merged snapshots are shown in the panel. Scale bar =20μm.

Re-analysis of *SARA* gene expression in normal skin compared to limited systemic sclerosis (lSSc) anddiffused systemic sclerosis (dSSc) patients, graphed out from data deposited as GSE9285, is shown (D).

Mann Whitney test **p<0.001 and Sidak's multiple comparisons test: *p<0.05.

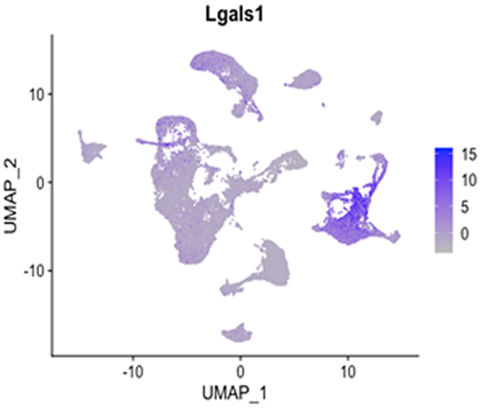

Fig. S6. *Lgal1* expression in pericytes-like cluster

Expression distribution of *Lgal1* represented by feature plot is shown and cluster 12, a pericyte-like cluster, expresses the highest level of this gene.

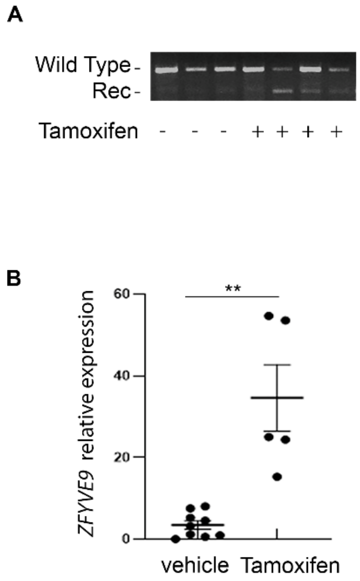

Fig. S7. ***Cre^{ERT2}*** **activation and** ***ZFYVE9*** **expression after Tamoxifen injection.**

Genotyping results of the *PDGFRβ-Cre^{ERT2};Z/EG;/$SARA^{Tg}$* mice with or without tamoxifeninduction are shown (A).

ZFYVE9 gene expression in skin of *PDGFRβ-Cre^{ERT2};Z/EG;/$SARA^{Tg}$* mice withor without tamoxifen induction is shown (B)

1. Livak KJ, and Schmittgen TD. Analysis of relative gene expression data using real-time quantitative PCR and the 2(-Delta Delta C(T)) Method. *Methods.* 2001;25(4):402-8.
2. Bonnardel J, T'Jonck W, Gaublomme D, Browaeys R, Scott CL, Martens L, et al. Stellate Cells, Hepatocytes, and Endothelial Cells Imprint the Kupffer Cell Identity on Monocytes Colonizing the Liver Macrophage Niche. *Immunity.* 2019;51(4):638-54 e9.
3. Browaeys R, Saelens W, and Saeys Y. NicheNet: modeling intercellular communication by linking ligands to target genes. *Nat Methods.* 2020;17(2):159-62.
4. Cao J, Spielmann M, Qiu X, Huang X, Ibrahim DM, Hill AJ, et al. The single-cell transcriptional landscape of mammalian organogenesis. *Nature.* 2019;566(7745):496-502.
5. Qiu X, Mao Q, Tang Y, Wang L, Chawla R, Pliner HA, et al. Reversed graph embedding resolves complex single-cell trajectories. *Nat Methods.* 2017;14(10):979-82.
6. hTraag VA, Waltman L, and van Eck NJ. From Louvain to Leiden: guaranteeing well-connected communities. *Sci Rep.* 2019;9(1):5233.
7. Trapnell C, Cacchiarelli D, Grimsby J, Pokharel P, Li S, Morse M, et al. The dynamics and regulators of cell fate decisions are revealed by pseudotemporal ordering of single cells. Nat Biotechnol. 2014;32(4):381-6.
8. O. h. E, H. Wu, Y. Muto, E. L. Donnelly, F. G. Machado, L. X. Fan, et al. A conditionally immortalized Gli1-positive kidney mesenchymal cell line models myofibroblast transition. Am J Physiol Renal Physiol. 2019; 316 (1) 63-75.
